# New recommender system evaluation approaches based on user selections factor

**DOI:** 10.1016/j.heliyon.2021.e07397

**Published:** 2021-06-27

**Authors:** M. Kshour, M. Ebrahimi, S. Goliaee, R. Tawil

**Affiliations:** a Faculty of New Sciences and Technologies, University of Tehran, Tehran, Iran; b Faculty of Sciences, Lebanese University, Beirut, Lebanon

**Keywords:** Diversity, Evaluation, Human behavior, Newness, Novelty, Recommender system, Variety

## Abstract

Currently, due to the increasing importance of recommender systems (RSs), especially in the fields of social networking and e-commerce, these systems represent one of the most interesting subjects in computer programming. Although many research reports have previously been published in this subject area, because of lack of clarity regarding their algorithms or limited comparisons with the literature, most of them are difficult to extend for similar applications in the future. Therefore, in the present study, we have attempted to improve two novel RS evaluation measures (variety and newness) developed from previous evaluator rules (namely, diversity and novelty) based on human behavior so as to be more reliable and compatible with various developments in RSs. The new rules provide higher weighting for suggestions and respect for users' behavior and can be used in place of diversity and novelty rules with better precision and centralization, by 22.54% for variety and by 14.84% for newness. In addition, we aim to use the developed measures to improve new RSs and support better comparative analyses in this field in the future. This contribution is expected to facilitate better RS research and competition, especially in the social networking domain.

## 1 Introduction

Approximately 250 million tweets and close to 15 billion messages are written every day [1]. The Telegram reports 200 million monthly active users, and more than 500 thousand new users join this service daily [2], and Facebook's daily active users numbered 1.4 billion on average in December 2017 [3].

Many surveys have been performed in this domain in the last four years [4, 5, 6, 7, 8, 9, 10, 11, 12, 13, 14, 15, 16, 17, 18, 19], and they have summarized most recommender system (RS)-related fields and provided good starting points to discover additional advantages and disadvantages. A summary of these surveys is presented in the next four paragraphs.

A comparison between these surveys and previous ones (before 2015) reveals the broad and fast development of RSs, especially in the last five years. The enormous expansion of social networking databases was the main cause of this occurrence. Accordingly, additional research works are now needed to enable further development in the RS field and facilitate the scientific utilization of these databases, for instance, to produce important and useful information. The means of producing such information in the RS field can be summarized by three words: detection, prediction, and suggestion.

RS learning provides a method of filtering and analysis that aims to simplify or clarify a subject to support easier diagnosis and decision-making by users or decision-makers, thereby reducing the cognitive load on users or system managers and introducing quality control. In general, the components of an RS can be divided into three types depending on the information source: (1) predictions based on saved databases and metadata of the contents, (2) context based on user/item actions, and (3) demographics based on user/item information and relations. For any particular application, the targeted data and the RS method used depend on the type of information that needs to be produced.

In brief, RS development starts with the filtering and analysis of historical data followed by an analysis of user/item activities. The RS makes comparisons based on user activities (collaborative filtering of user-user similarity), item activities (collaborative filtering of item-item similarity), graph-based detection of user social relations in social networking systems (e.g., LinkedIn, Facebook and Twitter), and model-based methods through the addition of new algorithms to the main RS methods, such as combining software engineering, machine learning and statistics.

As previously stated, a summary of interesting RS-related points has been derived from previous surveys [4, 5, 6, 7, 8, 9, 10, 11, 12, 13, 14, 15, 16, 17, 18, 19] as well as many evaluation and analysis methods to achieve this article's goals. Focusing on social networking, especially RS-related articles, we have observed that the differences between different evaluation and analysis methods can be very large, thus generating obstacles to the implementation and development of researchers' ideas.

As reported in [6], in some articles, no guidance is offered regarding which of the used approaches might be the most promising one. In other words, it is not possible to determine the most effective recommendation approach because too little information has been provided by the authors. Remarkably, 22% of the reported approaches have not been evaluated based on the existing evaluation rules at all, and of the remaining approaches, 20% have not been evaluated against a baseline, while most of the others have been compared only to simple baselines and not to other competing approaches [6].

In addition, the majority (71%) of approaches have been evaluated only in offline evaluations (using offline datasets); however, results based on such datasets do not allow reliable conclusions to be drawn regarding how the evaluated approaches might perform in real-world RS applications, which raises questions about the significance of these evaluations. Notably, only 8% of approaches have been evaluated online in real-world RSs with real users [6]. Moreover, the scattered nature of published RS papers makes it difficult to collect or extend previous research works, pushing us to search for better RS development conditions, starting with a global view of analysis and evaluation approaches. Thus, the current research aims to develop two rules for evaluation, based on metrics of diversity and novelty. These metrics can support better and deeper analysis of previous and future RSs based on the basic algorithms for top-N and K-nearest neighbor (KNN) RSs [5], two classes of RSs that have been widely tested and used before and can be easily implemented in many programming languages. In addition, this research uses online databases [41] that have been modified by users throughout the world and are available as open-source datasets. Because RS evaluation rules constitute a major focus of this contribution, and the idea of merging is considered very interesting and effective in RS development for improving user satisfaction and system utility [14], the two evaluation rules proposed in the current research are developed by merging existing rules with concepts of human behavior.

Two main achievements are targeted in the research: developing two new evaluation rules in order to show better results in terms of RS suggestions diversity and novelty evaluation, and improving RS suggestion effectiveness through a real-time evaluation system of suggestions and user selections using the two new rules. Previous evaluation rules have focused on the evaluation extracting of a single RS capability (novelty, diversity, accuracy, etc…), regardless of other parameters, especially those related to detecting user interactions with the presented predictions. The new rules allow the users' acceptance of the quality of RS services to be evaluated through a calculation of their tendencies toward diversity or novelty, in order to attract them to spend more time using RSs, and provide better user satisfaction. Both the old and new rules can run outside or inside the RS algorithms, but the main difference is that the old rules are designed to improve RS service based on the past expected quality or level of evaluation, whereas the new rules can provide real-time values reflecting a user's accepted level of quality or level of evaluation, and thus, can provide better real-time suggestion improvement in order to achieve a higher attractiveness for users. The proposed system allows RS suggestions for a user to be incrementally improved on the basis of incremental changes in user practice over time. This is achieved by discovering users' behaviors and their curiosity in recognizing and pursuing unexpected choices [29]. Furthermore, adding unexpected suggestions among the initial RS suggestions while managing the suggestion list based on user behavior may hold a number of benefits for many fields, especially in commerce. Previous diversity and novelty rules evaluate suggestions list at each RS prediction, but the new rules allow one of the RS's prediction capabilities (diversity or novelty) to be evaluated simultaneously with the user reaction to this capability with respect to the user's choices (user selections), and this helps to develop the suggestions' effectiveness at every user selection. The new rules are currently limited to evaluation based on users' rankings and selections, but it may be developed and tested for other parameters in future research. Additionally, although the old and new rules have been developed to work with most types of RSs, they have not been tested with all possible types; thus, we must consider the possibility of failure with some RSs.

The baseline of the research developments is summarized by providing more effective evaluation rules with new parameters that help inform new evaluation concepts and enhance RS abilities at the same time. This baseline is based on the previously mentioned surveys that have investigated the development of evaluation rules, enabling a comparative study of the capabilities of some similar evaluation rules and reference points from previous articles, especially: novelty, diversity and user behavior. As shown, new evaluation rules are developed, compared with previous similar rules, and tested with two types of RSs and two types of datasets, and finally, approaches are presented for the practical utilization of the newly developed rules outside and inside RSs.

## 2 Literature review

Since evaluation development is the main goal of this research, to lay the foundation for the implementation of new evaluation rules, all of the related work in this part is presented as an introduction to the intentions of the paper. The review starts with surveys to help create a global view, providing an introduction to the RS domain.

### 2.1 RS and evaluation

One previous survey on RSs [7] presents brief descriptions of various types of recommendation techniques. It also discusses feedback techniques for RSs. Its main conclusion is that combining RSs with machine learning and natural language processing can support the development of powerful and efficient RSs. Another RS survey [16] provides a brief review of three generations of RSs. The similarity measures and evaluation metrics used in these generations are also discussed.

**Figure 1:**
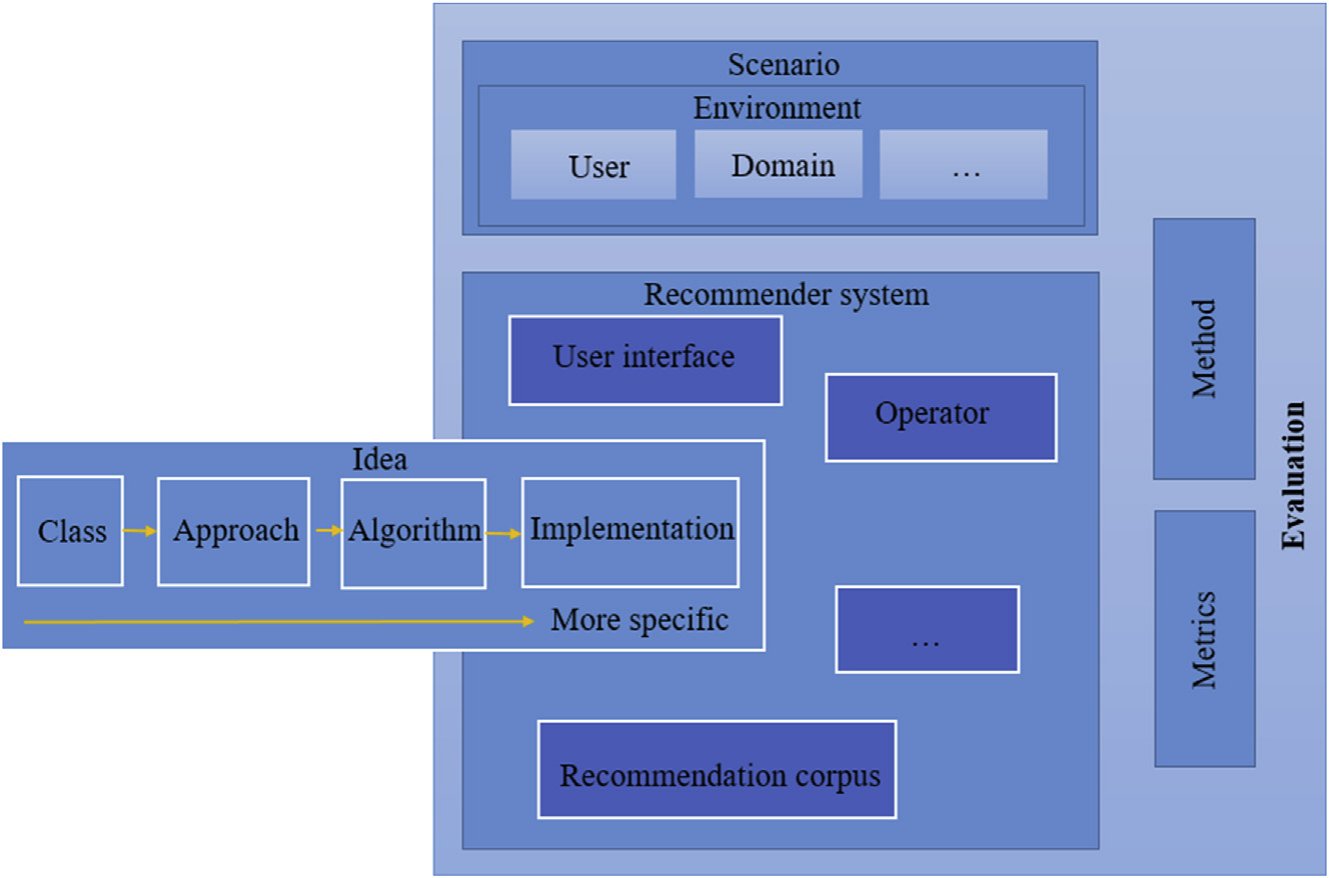
Role of evaluation in RS implementations [24].

This survey further presents the advantages and limitations of different types of RSs in each generation. Moreover, the quality of various techniques, algorithms and procedures is evaluated in terms of effective prediction and recommendation. The evaluation metrics used for this purpose can be classified into different types, as shown in Figure 1 [27]. Figure 1 shows the general steps of RS implementation and the role of evaluation, clarifying that previous evaluation rules have been separate from the main RS algorithm and have focused simply on evaluating its outputs; in other words, the existing rules are not used to change or develop RS frameworks but only to show their capabilities.

### 2.2 Evaluation definition

The article [27] summarizes the main existing evaluation metrics, for which the corresponding rules are focused mostly on accuracy. However, these metrics have not been adopted in some recent articles because of the undesirable influence of high accuracy on users’ activities; instead, other rules have been provided, such as rules based on diversity and novelty. A survey focusing on technology-enhanced learning (TEL) [8] provides some evidence that there are still many challenges facing the evaluation of RSs in the context of TEL. The evaluation of RSs is a complicated process, in which complex research questions need to be broken down into smaller and more practical subquestions. That survey presents important charts showing the changes in the predominant RS types per year as well as the tendency to use more extensively evaluated systems. The results indicate important differences in the sizes of the evaluations needed for different RS algorithms depending on the RS type. The survey also presents charts showing the effects of the growth of RS evaluations on system characteristics and the differences in implementation between different RS types. Ideally, every RS type should be studied individually to understand the corresponding changes over time, but a common finding among the different types of RSs is the considerable influence of the development of new evaluation approaches on their characteristics. Overall, evaluation development should lead to higher usability and availability for a wide variety RS types, yielding new rules can run either as a part of an RS algorithm or as an independent means of filtering suggestions, which is the goal of the current research.

### 2.3 Human behavior

A paper on recommendation methods for big data applications [20] presents a newly developed method of personalized recommendation, which has been executed on Hadoop to make the technique more effective and scalable. The results show that the proposed recommendation method is superior to current traditional methods. Its major advantages derive from its scalability and greater efficiency compared to traditional methods. Two evaluation approaches are tested in that paper, and a comparison is presented. The most interesting aspect of that study in regard to the current article is the benefit of incorporating personalized parameters (human behavior). The new evaluation rules presented here can support the consideration of human behavior regardless of the RS type used through filtering of the final suggestions generated by the RS based on these rules, which is also the approach adopted in the current article. A previous study on e-commerce recommendation [21] has suggested a new clustering technique based on a latent class regression model (LCRM). In essence, it is able to consider both general ratings and feature-level opinion values (as extracted from textual reviews). In the testing stage, the authors tested the proposed recommender algorithm, including a non-review-based technique and non-LCRM-based variations, on two true datasets. The algorithm is similar to the applicability approaches proposed for the new evaluation rules in the current research. Before delving more deeply into evaluation methods, to show the importance of the scope of the presented research, which focuses on the merging of evaluation methods and human behavior, we remark on the effectiveness of considering popularity (users’ ratings) in recommendation [39], as popularity is still the main factor considered in the generation of successful suggestions.

### 2.4 Evaluation challenges

In a previous RS evaluation paper [22], evaluation metrics are chosen and redefined to evaluate a new RS. The paper concludes that based on previous studies, in addition to accuracy, a variety of other metrics should be taken into account when evaluating RSs, and many RSs need to be tested to demonstrate any proposed evaluation approach. It is deduced that for every type of evaluation, a suitable implementation should take the majority ratio of results and discard what remains. In other words, for the evaluation results of RS, it is better to perform based on the majority ratio of evaluations. This explains the current research innovations, in which proposed rules have been tested based on overall analysis tags (averages). A previous survey on RS evaluation [15] consists of a summary of interesting papers that propose new RS evaluation approaches and strategies. In addition, it presents the relationships between concepts, categorizes methods according to their objectives, and suggests potential future topics of research related to user satisfaction. This survey is an interesting summary of the RS evaluation domain and a launching point for our research. Such surveys highlight the limitations of previous evaluation approaches and inspire the development of the novelty rule proposed in the current research, which focuses on the distance *d(x,j)* (distance-based item novelty [34]) between a predicted item and other items in the prediction list based on a similarity equation for determining the distance value, and the diversity rule also proposed in the current research, which focuses on the distances *d(i,j)* between all predicted items. These evaluations do not relate RS results to user satisfaction; for this reason, the new rules use two existing concepts but expressed in new functional forms that include a human behavior parameter related to user selections and are applied only to the final RS suggestion list, thereby reducing the number of items evaluated for novelty and diversity, to produce feedback on user behavior. Further explanations are given in the experimental testing section of this paper.

#### 2.4.1 Randomness challenge

A decentralized RS paper [23] compares centralized and decentralized user-based and item-based collaborative filtering (CF) systems. It develops a new user-based random walk approach specifically for decentralized systems that is designed to handle sparse data. Simulations on the MovieLens dataset, with 10,000,000 ratings, show a wide range of sparsity. Consequently, the new algorithms outperform other decentralized CF schemes and perform better than similar item-based approaches in peer-to-peer (P2P) recommender algorithms. In the current research, the benefit of randomness is also discussed, and new rules are developed for reducing sparsity through better evaluation of RS suggestions. A rating-based RS paper [24] proposes a system that uses clustering and a random forest model in a multilevel strategy for predicting recommendations based on users’ ratings while targeting mindsets and current trends. The results are evaluated with the help of the root mean square error (RMSE), and feasible performance is achieved.

#### 2.4.2 Human behavior challenge

Another article studies user participation in a course RS [25] investigate the influence of human behavior in such an RS with a special working mechanism. The present study addresses human behavior evaluation with the aim of taking advantage of the corresponding results in any type of RS. A paper on digital records of human behavior [26] focuses on the monitoring of users' behaviors in order to understand their thought patterns using psychological techniques, with the ultimate goal of increasing user trust in RS services. Similarly, the present work develops new approaches for evaluating user behaviors to support the development of user profiles and the generation of suggestions that more closely adhere to users’ particular ways of thinking. A survey of the evaluation of RSs from the user perspective [14] presents a brief study of different types of interactions between users and RSs as well as the evaluations possible from this perspective. The authors classify different types of user interfaces to help system designers develop more effective RSs that offer better user satisfaction. The results show that human behavior is the best and most effective factor determining user satisfaction and high utility of RSs.

#### 2.4.3 Improving challenge

Developing new concepts of diversity and novelty evaluation, article [31] uses novelty as a parameter (degree) for guiding diversity variations. The underlying idea of this article is the most similar to that of the current research, in which a human behavior parameter (user selections) is used as a main variable for novelty and diversity evaluations in the context of a new concept of applicability, with the goal of providing better user satisfaction and utility. We can introduce the current research variety approach as a type of individual diversity since it targets to provide better suggestions based on individual perspectives, but the differences exist in the methods or approaches used by each study, like a providing multiple drawbacks (integrated decision-making model) associated with the KNN RS algorithm [36], or re-ranking model improves the diversity by employing personalized Determinantal Point Process [37], or re-rank the list of Top-N items predicted by a recommendation algorithm [38]. These methods have some similarities with current research in the idea but they focus on ranking improvement or RS algorithm development in order to improve individual suggestions.

### 2.5 Research features

#### 2.5.1 Offline testing

Based on a paper comparing online and offline evaluations [35], offline evaluations may have some predictive power in cases in which users' activities will have equal or no impact on the RS algorithm. The new rules based on the metrics developed in the current research (variety and newness) are tested through an offline evaluation of the suggestions selected by users with respect to the suggestions offered by other RSs, which are not related to the possible real-time changes in online datasets, whereas previous similar rules (based on diversity and novelty) have been used to evaluate RS recommendation lists, which may change during online testing. Therefore, in the current case, the users' activities are expected to have equal impact under the new evaluation approaches because they may change the recommendation list but will not change the evaluation of the users’ selections.

#### 2.5.2 Human factor

Articles related to human behavior have shown how it is possible to use important related factors in the development of RSs. The challenge is to add appropriate human behavior parameters into RS frameworks or evaluation rules. The current research focuses on developing a novelty rule (at the level of the recommendation list [15]) and a diversity rule by implementing new formulas that include a user selection parameter and can be used to evaluate, for each user, her/his tendency to select RS suggestions that show high diversity or unexpectedness (novelty).

#### 2.5.3 Clarity of approaches

Surveys have revealed problems that need to be addressed in RS evaluation, such as testing user trust, the effect of high accuracy, privacy (regarding behavior and emotional evaluations), and many others. They can also help search for the best testing methods to improve them.

A previous survey [16] clearly focuses on the evaluation problems evident in many RS articles, as well as the poverty of clear algorithms therein. Other surveys [8, 15, 16] investigate, in different manners, the problem of a lack of evaluation in many articles. They argue that there are large disparities in accuracy, testing, analysis, and unified evaluation types used in previous articles, indicating problems with development and implementation. Consequently, it is difficult for new researchers to fully comprehend or take advantage of the benefits of the methods presented in many previous articles.

The present report considers a wide review of previous work on RSs in order to find the most helpful working principles for RS implementation. In addition, simple testing of some widely used open-source RSs is performed to determine the benefits of working with certain standards within a framework to help future researchers easily understand, test, compare, evaluate and develop common research projects.

### 2.6 Summary

The related works section has presented a review of several interesting papers, summarized in Table 1, which were helpful in guiding the underlying ideas and development of the presented research. Table 1 represent a summary of most related work articles that can help future research for easier developments. For these related works, we sequentially display the definitions of the RSs, the evaluation definitions and types, their effects on the systems and their importance in system development, simulation and analysis. One important point to note is the large influence of evaluation approaches on RS development, especially in terms of accuracy, diversity and novelty. Various types of evaluation approaches have been applied, although an absence of evaluation is still a problem for some systems [6], and many RS evaluation methods still show a need for further development. For this purpose, the current study attempts to delve deeply into several problems encountered in RS evaluations, such as the addition of new parameters or even scientific disciplines (data mining, decision-making, etc.).

**Table 1:** Summary of works related to RS evaluation.

Year, author, reference	Main subject	Proposed method/algorithm	Dataset(s)	Evaluation criteria	Advantages	Limitations
(Meng, Dou et al. 2014) [17]	New user-personalized RS approach	User-based CF algorithm	Hadoop	MapReduce parallel processing paradigm	New keyword-aware service recommendation method. Improved accuracy and scalability	Users' personalized requirements. Keywords are used to indicate users' preferences
(Chen and Wang 2013) [13]	Novel clustering method based on an LCRM	Establishment of a connection between an active buyer and a cluster of reviewers by revealing the interrelevance of their preferences	Digital camera dataset and laptop dataset (www.buzzillions.com)	Performance of approach in terms of increasing the system's recommendation accuracy, using Kendall's tau, the hit ratio and the mean reciprocal rank	Able to consider both overall ratings and feature-level opinion values (as extracted from textual reviews) to identify reviewers' preference homogeneity. Increased recommendation accuracy of the system	Focused on e-commerce, for which there exists a special implicit community composed of product reviewers. Tests on two real databases and compares with two similar RSs
(Silveira, Zhang et al. 2017) [12]	Survey of RS evaluation methods	None	None	None	Traditional concepts of evaluation are explored. Studies the relationships between concepts, categorizes methods according to their objectives, and suggests potential future topics of research related to user satisfaction	Evaluation methods. Focus on satisfying user requirements
(Ajesh, Nair et al. 2016) [21]	New prediction algorithm	A new system using clustering and a random forest model in a multilevel strategy for predicting recommendations	Hetrec2011-movielens-2k for testing user ratings while targeting their mindsets and current trends	RMSE	The processing performance of the random forest model is satisfactory for a large number of labels. The clustering accuracy is good, and the number of iterations needed to converge can be reduced by means of the initial seeding	Specific for a particular application. Specific new prediction method
(Farzan and Brusilovsky 2011) [22]	Investigation of the impact of encouraging user participation in the context of Course Agent, a community-based course RS	Course Agent applies an incentive mechanism to convert user feedback into a self-beneficial activity	Two series of evaluations conducted in the School of Information Sciences at the University of Pittsburgh	Monitoring user reactions	An incentive based on personal needs motivates self-deception, which causes a positive rating bias in this case. Different incentive mechanisms trigger different side effects and are subject to different problems	New mechanism and study of the impact of user behavior. Impact of used mechanisms on user behavior in RSs has not been studied sufficiently
(Pu, Chen et al. 2012) [11]	Survey on the evaluation of RSs from the user perspective	Investigation of three crucial interaction activities between user and system: the initial preference elicitation process, the preference refinement process, and the presentation of the system's recommendation results	None	None	Evaluates design methods that augment an RS's ability to help users find items they truly prefer and form trust with the RS through system transparency, control and privacy preservation mechanisms. Provides a set of design guidelines that serve as useful suggestions to scholars and practitioners concerning the design and development of effective RSs	Survey-specific evaluation methods based on user interactions. Many researchers have recently started investigating system effectiveness and evaluation criteria from the user perspective
(Mendoza, M & Torres, N. 2019) [28]	New novelty and diversity approaches	Diversity approach based on the degrees of novelty of the elements that make up each item	MovieLens, MovieTweetings	Degrees of novelty of the elements that make up each item	More consistent and interpretable results, reducing the impact of popularity bias on evaluation. Development of new features for previous evaluation rules will improve RS evaluations	Specified two evaluation methods. New perspective on evaluation development
(W. Luan et al. 2017) [27]	Method for recommending points of interest	Collaborative tensor factorization technique	Weibo and DianPing websites	RMSE, MAE	Better accuracy. Mathematical analysis for developing new concepts	Accuracy evaluation with human behavior. Focuses on the development of an RS framework
(Castells, Pablo, Neil J. Hurley, and Saul Vargas. 2015) [31]	Study of a formal characterization of different aspects of RS novelty and diversity from the end-user viewpoint	Incorporation and generalization of metrics reported in three previous articles and derivation of a new one	MovieLens	Novelty and diversity	New approaches are introduced in a generalized way based on easy-to-configure terms involving any metric supported by our scheme. Preliminary experiments confirm hypotheses and provide initial observations on the behavior of the different metric configurations	Specific development based on diversity and novelty metrics. Limited experiments. Room remains for deeper examination and additional empirical studies involving specific tasks and scenarios to provide further insight into the qualities of the metrics for different purposes

## 3 Methodology

The study's main proposed idea is to improve a solution for adding fundamental factors that affect user satisfaction and RS usability, like human behavior, by developing new evaluation rules (newness and variety) based on previous similar rules (novelty and diversity) that consider a user behavior parameter. The idea is also to discover the ability to enhance RS suggestions by adapting suggestions based on new rules for real-time testing of user behavior (selections). Besides the above, there are challenges solved by the new evaluation system presented below.

a) Considering the worst possible case by studying the effects of unexpected suggestions on both new and previous evaluation rules (e.g., diversity and novelty).
b) Controlling the percentage of unexpected (random) suggestions based on an analysis of user satisfaction through the development of new RS evaluation rules.
c) Proving the benefits of adjusting the different types of suggestions presented to achieve better diversity and novelty.
d) Ensuring that every RS can generate predictions for a user such that the suggestions will remain with in an accepted range of the user's evaluation degree by controlling the ratio of unexpected suggestions based on the detected type of user behavior, according to an analysis of the user's selections (diversity-seeking or novelty-seeking type).

**Figure 2:**
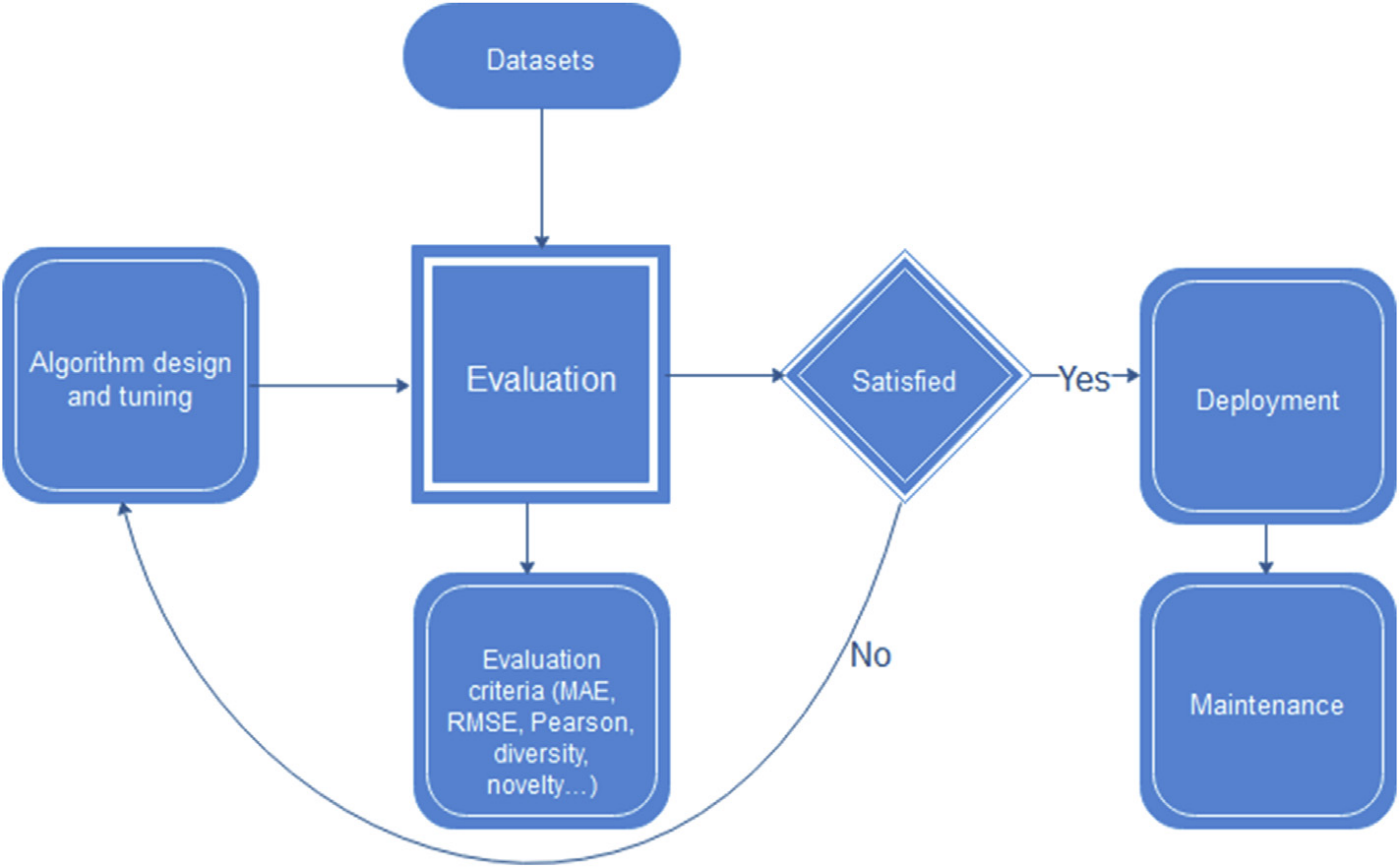
RS evaluation cycle.
• The addition of random suggestions reduces the relative number of RS-predicted suggestions and thus carries a risk of losing any potential benefits of adding random suggestions by affecting the RS evaluation accuracy. However, the new rules solve this risk by assisting in managing and administrating the worst cases of unexpected diversity and novelty.

We additionally note the following:

• The old usage system of previous evaluation rules (Figure 2) cannot provide real time behavior detection as the new evaluation rules can because they focus mainly on suggestion evaluation. The current research investigates the addition of random (unexpected) suggestions and attempts to control how such suggestions are added based on a human behavior parameter (user selections) and evaluations of the suggestions (in terms of diversity or novelty).

Testing is performed to determine how best to add a percentage of unexpected suggestions that will be acceptable to the user based on the new evaluation rules and considering the worst case for suggestions (random suggestions) to increase the unexpectedness, diversity, and novelty in the controlled system. This will also increase users' curiosity, allowing them to discover new suggestions in the system database outside of their specific targets, in combination with mechanisms for controlling what suggestions are presented based on new rules to avoid compromising user acceptance of the unexpected suggestions. In other words, the new rules are designed to detect a user's levels of acceptance of diversity and novelty.

The new rules provide the opportunity for any RS to adopt a human behavior parameter and evaluate diversity or novelty with respect to this parameter. The main goal of enhancing diversity or novelty is to improve user satisfaction; thus, when the system can calculate corresponding evaluations from the users' perspective, it will be able to better improve user satisfaction and its own attractiveness to users. For this reason, the new rules offer more useful perspectives for RSs. Additionally, they provide better centralized results that support enhanced control of the risk to user satisfaction presented by increased diversity or novelty and more comprehensive quantitative results (better comparison between the diversification and novelty of suggested items). In this way, it becomes possible to more effectively compare items with similar evaluation values or even to compare different users, if necessary (for example, comparing two users’ behaviors). In addition, the new rules allow the promising possibility of replacing the human behavior parameter used here (user selections) with other parameters, such as the time spent using the system depending on the degree of diversity of the suggestions, which is a new challenge that can be addressed in future research.

## 4 Proposed approaches

**Figure 3:**
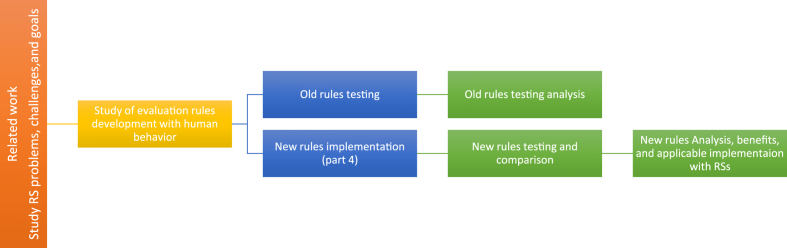
Research implementation steps.

Figure 3 summarizes the research's main study and implementation steps, starting from the study of RS in previous articles up to the applicable testing and analysis in the last research steps.

**Figure 4:**
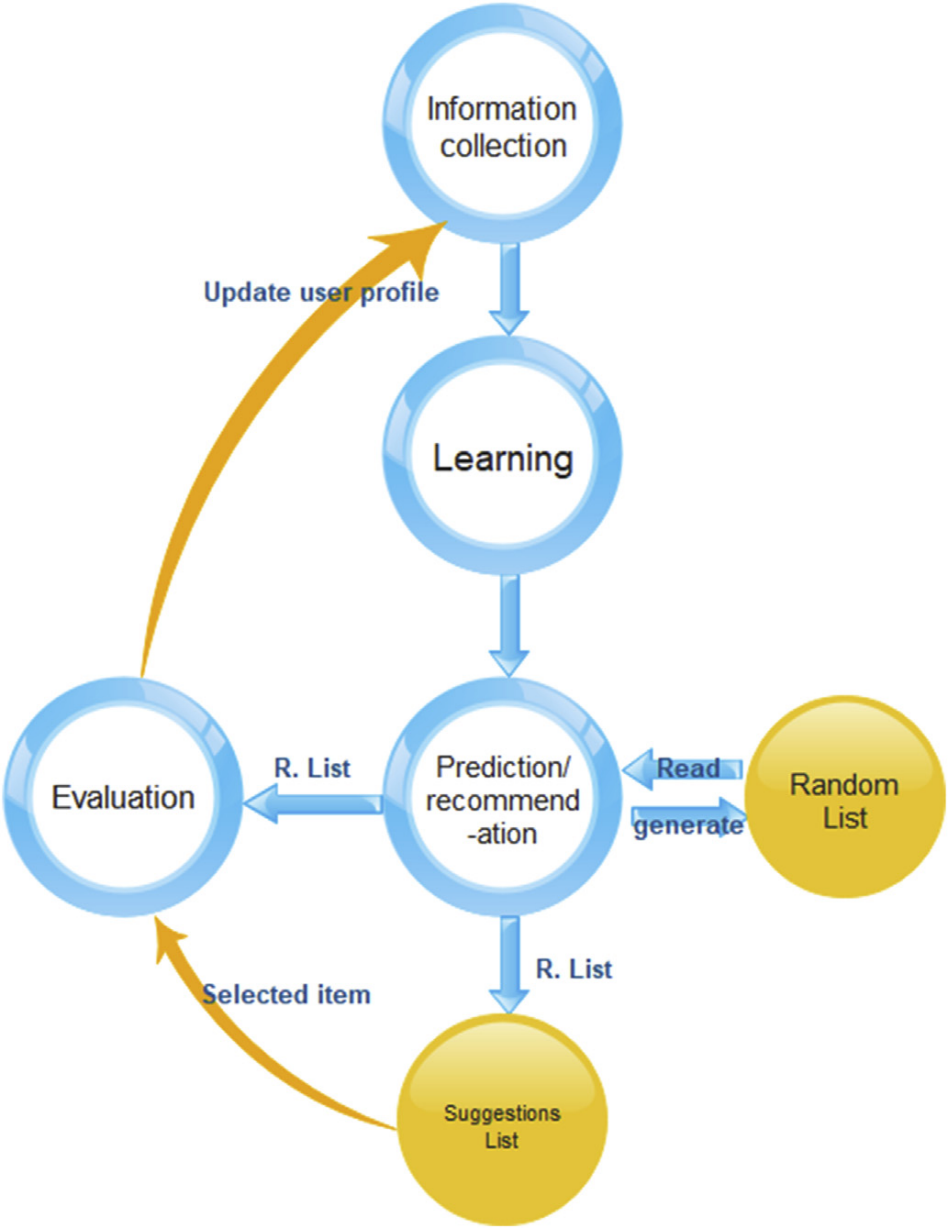
Recommendation process.

In Figure 4, information collection is the phase corresponding to user profile/model construction and maintenance. Learning is the phase in which users’ information is analyzed and studied based on the established evaluation methods and feedback returned from user activities. Prediction and recommendation is the phase corresponding to the prediction and recommendation of suggestions based on the type of RS that is implemented [32].

### 4.1 Research model and algorithms

Evaluations can be implemented without any fundamental changes in the initial RS algorithms [33]; only a simple change is made to allow the ratio of random suggestions to be set in accordance with the results of the new evaluation rules that are saved in a user's profile (Algorithm 1). Algorithm 1 steps: step 1: read the average x of evaluation degrees of a user from the user profile (which represent the user acceptance ratio of unexpected items), step 2: generating the prediction list by the RS, step 3: based on x value, the system will know the appropriate number n of random items, that should be generated and (step 4:) added to the final suggestions in place of n predicted items.

**Table:** 

Algorithm 1: Recommender system
Input: Average evaluation degrees x
Output: Generate prediction list [30]
Based on x, generate n items randomly
Replace n predicted items in the list of suggestions with the n random items

Figure 4 explanation: The new rules are added as a new class to the RS algorithm, and the RS sends its list of suggestions (recommendation list, R.list), containing the required percentage of random suggestions for the user, to the evaluation class. Then, a set of item identification (ID) numbers is generated by running a random function consisting of a loop that runs a random command n times, where n is determined based on the results of user behavior evaluation saved in the user's profile; for example, for the generation of 10 suggestions with a 20% newness value, the random function will generate a number of random item IDs equal to 20% of the final list of suggestions generated by the RS, i.e., n = 2, to replace two items in this list (list of 10 items in the current research). Then, the evaluation class evaluates the item selected by the user based on its position with respect to the other nine suggestions in the list (i.e., evaluates the level of diversity or novelty represented by the selected item within the recommendation list) and sends an update to the user profile (send the selected item evaluation result), which consists of the newly evaluated degrees and the average of all evaluated degrees. The RS recommendation list is generated based on this average degree in the sense that it is used to determine the appropriate percentage of random suggestions in the final list. If desired, the selection of random suggestions can also be replaced by an item list that has been prepared in advance (e.g., advertisements).

Many methods have been used in RSs or in previous evaluation approaches for calculating the distances between items or users. In this paper, the distance (d) used in the evaluation rules is the cosine similarity (Eq. 1) calculated from an item rating dataset [16] downloaded on 2-1-2019 (version 2018). More specifically, the proposed approach is tested on two rating-based datasets: the MovieLens dataset (containing 100004 ratings [0 to 5] generated by 671 users for movies chosen from a database of 352 movies) and the BookCrossing dataset (containing 1048576 ratings [0 to 5] generated by 278858 users for books chosen from a database of 271380 books). The ratings in the datasets serve as the basis for the predictions of the RS, and the new rules are evaluated based on the ratings for 10 items suggested by the RS [13].

(1) $$\cos\left(a,b\right)=\left(\sum_{i\in S_{a,b}}r_{a,i}.r_{b,i}\right)/\left(\sum_{i\in S_{a,b}}r_{a,i}^{2}.\sum_{i\in S_{a,b}}r_{b,i}^{2}\right)$$

where S_a,b_ is the set of items rated by users a and b, r_a,i_ is the rating given by user a to item i, and r_b,i_ is the rating given by user b to item i [16].

**Table:** 

Algorithm 2: New evaluation rules
Input: Recommendation list (R.list)
Output: Evaluate and update user profile:
while new selection
do
if item i selected
{
i degree = Evaluate i in R.list
Save new degree in the user profile
Calculate new average of user degrees
Save new average in user profile
}
end while

Algorithm 2 presents a simplification of how the new evaluation rules are implemented; Algorithm 2 steps: Step 1: read the suggestions list, step 2: wait if the user select an item, step 3: if an item is selected then calculate the degree of diversity or novelty inside the list of suggestions presented by the RS, step 4: save the degree value in the user's profile, step 5 and 6: calculate degrees new average and saved in the user profile. The average of all of the degree values calculated for the user in accordance with the new evaluation rules is the evaluation value needed for detecting user behavior. The variations of this average value reflect how the user's interactions with items in the R.list are related to the level of diversity or novelty of those items. No major change is made in the tested RS algorithm because the goal is to prove the ability of the new rules to be implemented either inside or outside the RS framework, allowing the proposed approach to be flexibly used with all RS types. Most of the experimental testing in this study is based on two RSs that serve as good baselines for RS development. The first is a top-N RS, which is a CF-based RS that uses a model-based technique to analyze the user-item matrix to detect the relations between items in order to generate top-N recommendations. The second is a KNN RS, which also uses the CF technique to analyze the historical ratings of users for items.

The new rules are developed on the basis of previous evaluation approaches [diversity (Eq. 2) and novelty (Eq. 3)] based on the testing (in MATLAB software) of many newly proposed functional forms that include a human behavior parameter in order to deduce better rule expressions, with the aim of ultimately providing better conclusions and better results than previous rules.

The number of tests performed in this research was greater than the number of tests for which results are presented in the next section (laboratory testing); the presented results are condensed because more testing did not yield new inferences, and the choices of 196 iterations of every test and 25 iterations in the applicability study are sufficient for proving the research goals.

The selected datasets [41] contain item IDs, user IDs and ratings. These datasets correspond to well-known databases that are available as open-source resources, have been widely tested in previous research, and are related to online RS operation. These characteristics are beneficial for future developmental research.

### 4.2 Approaches

The two new rules are basically extensions of the previous developed concepts of diversity and novelty. Diversity (div) is a concept that concerns the diversity of the items in the recommendation list [15]. Based on a previous survey [15] and using the basic diversity rule (Eq. 2) as the foundation of the variety rule developed below, we experimentally calculate the diversity of the prediction list L_u_ based on the distances d (i,j) among the items in the list to study the effect of decreasing the percentage of random suggestions on the previous diversity rule, where d (i,j) is the distance between two items i and j:

(2) $$\mathrm{div}\left(L_{u}\right)=\underset{i\in L_{u}}{\sum }\underset{j\in L_{u}}{\sum }d\left(i,j\right)$$

The concept of novelty generally concerns the presence of novel items in the recommendation list [12]. According to a previous survey [15], there are three main types of novelty:

- Lifetime level: The item is novel among all user experiences in the system.
- System level: The item is novel based on the user's consumption history.
- Recommendation level: The item is novel if it is not in the recommendation list.

In the same manner as for diversity testing, the variations in novelty (nov) with an increase in the percentage of random suggestions are experimentally studied, in accordance with Eq. (3), which serves as the foundation for the newly developed newness rule:

(3) $$\mathrm{nov}\left(L_{u}\right)=\left(1/\left|L_{u}\right|-1\right)\underset{j\in L_{u}}{\sum }\left(1-d\left(i,j\right)\right)$$

#### 4.2.1 Variety

Variety (Var) is a new concept for human behavior evaluation that depends on the types of items selected by a user. The old diversity-based evaluation rules evaluate the level of diversity of RS predictions, whereas the variety rule evaluates the level of diversity of the items selected by a user to *assess the user's preference for diversity in the suggestion list*. This value is used as a new entry in the user profile. In other words, it is used to discover users' behavior in regard to variety.

First, the degree of variety (V) associated with the selection of each item (s) is calculated (Eq. 4) to represent the diversity of the items selected from the suggestion list with respect to the other suggestions (L_k_) based on the distance (d) values between these items. An efficient and easy-to-use mathematical formula for variety (similar to the newness rule presented below) has been developed through deep analysis, and many experiments have demonstrated the efficacy of the given equations. The main goal is to test the position (diversity level) of the selected item relative to the other suggested items; therefore, the value of d, which represents the distance between s and another presented item for the user, is used. In this paper, *d* is calculated based on rating values, but the rating values could be replaced with other values (of another measurement parameter) if desired. The results would still exhibit the same logic for a new range of values.

In place of calculating the diversity between all predicted items (Eq. 2), we calculate only the diversity of the selected item (s) in the final suggested item list (Eq. 4), where $\sum_{i}^{k}d\left(s,i\right)$ is the sum of the distances *d* between the selected item *s* and the other suggested items *i*. The distance between *s* and *i* represents the possibility that the suggested item i will also be selected if *s* is selected. However, the calculation of the distances with respect to s alone is not sufficient to extract the correct variety (diversity based on user choice) because of the unhelpful results produced by such an experiment. Therefore, the rule also needs to consider the distances from the opposite perspective, as represented by $\sum_{i}^{k}d\left(j,s\right)$, which corresponds to the total distance *d* between all suggested items j and the selected item s. Here, the distance between *j* and *s* represents the possibility that *s* will be chosen if one of the other suggested items, *j*, is selected. As shown in the diversity rule (Eq. 2), division by *2k* is adopted to simplify the value of the result to be closer to the domain of the distances, and the square root is similarly taken to provide a clearer result (similar to the relation between the mean absolute error (MAE) and the RMSE in the accuracy rules [24]). Further explanations of such distance calculations are provided in previous surveys [4, 5, 6, 7, 8, 9, 10, 11, 12, 13, 14, 15, 16, 17, 18, 19].

Finally, the degree of variety for one selection among *k* suggestions is calculated as follows:

(4) $$V_{s}\left(L_{u}\right)=\sqrt{(\sum_{i}^{k}d\left(s,i\right)+\sum_{j}^{k}d\left(j,s\right))/2k}$$

However, the calculation of the Vs degree for one selection is not sufficient to correctly deduce the user's behavior (because user decisions are not always rational); instead, the final variety evaluation is calculated as the average of all calculated variety degrees (n) in a specified time slice (Eq. 5), i.e., the sum of the set of calculated variety degrees divided by the number of these degree values.

Thus, the degree of variety of n evaluated items for user u is calculated as follows:

(5) $$Var{\left(V_{n}\right)}_{u}=\left(\overset{n}{\underset{0}{\sum }}V_{n}\right)/n$$

The time slice should be chosen depending on the system managers’ assessment of the optimal number of degrees and should be calculated with *Var(V*_*n*_*)*_*u*_ or the best time division to ensure the quality of the evaluation results (the same also holds for the newness evaluation).

#### 4.2.2 Newness

Newness (Newn) is the second new concept for human behavior evaluation with respect to the types of items selected by the user. The old novelty evaluation rules evaluate an RS based on the recency of its predictions, whereas the newness rule evaluates the novelty of the items selected by a user in accordance with their recency in the suggestion list to yield a value *indicating the user's preference for or interest in novelty in the list of suggestions*. This value is also treated as a new entry in the user profile and is used to discover users' behavior in regard to newness.

Similar to the implementation of the variety rule, an efficient and easy-to-use mathematical formula for the newness rule has also been developed through deep analysis, and many experiments have demonstrated the efficacy of the given equations. As mentioned above, the main goal is to test the position of the selected item relative to the other suggested items; therefore, the value of d, which represents the distance between s and another item presented to the user, is used, where d is calculated based on rating values. However, as in the case of the variety rule, the rating values could be replaced by other measurement values, and the results would still exhibit in the same logic but with a new domain of the result values.

In place of calculating the novelty between all predicted items (Eq. 3), we calculate only the novelty of the selected item (s) in the final suggested item list (Eq. 6). First, the degree of newness (N) for each item selection is calculated. Eq. (6) presents the novelty of the selected item s with respect to the other suggestions L_k_ in the suggestion list L_u_ based on the distance between s and each other item in L_k_ (here, *k* = number of suggested items - 1); note that for this rule, it is necessary to consider the distances only from the perspective of the selected item (as in the novelty rules used in previous studies [24]).

Finally, the degree of newness of a selected item s is calculated as follows:

(6) $$N_{s}\left(L_{u}\right)=\sqrt{\left(1/\left|L_{k}\right|\right)\sum_{i}^{k}\left(1-d\left(s,i\right)\right)}$$

where *d(s, i)* is the distance between the selected item s and another suggested item *i*, which represents the possibility that item *i* will be selected if s is selected.

Then, the final newness evaluation is calculated as the average of all newness degrees of a set of individual selections (Eq. 7).

Thus, the degree of newness of m evaluated items for user u is calculated as follows:

(7) $$Ne\text{wn}{\left(N_{m}\right)}_{u}=\left(\sum_{0}^{m}N_{m}\right)/m$$

### 4.3 Testing preliminaries

For every test in the experimental testing results presented in the next section (Figures 6, 7, 8, 9, 10, 11, 12, 13, 14, 15, 16, 17, 18, 19, 20, 21, 22, 23, 24, 25, 26, 27, 28, 29, 30, 31, 32, 33, 34, 35, 36, 37, 38, 39, 40, 41, 42, 43, 44, 45, 46, 47, 48, 49, 50, 51, 52, 53, 54, 55, 56, 57), 196 iterations are presented, with the proposed time slices consisting of every 28 iterations. Accordingly, seven sets (phases) of 28 iterations are implemented as time slices, with the percentage of random suggestions increasing in every phase (from 0 to 60%). From the first slice to the seventh slice, the percentage of random suggestions (worst case) or diversity percentage (Figures 56 and 57) increases in increments of ten percent. Each pair of figures (Figures 6, 7, 8, 9, 10, 11, 12, 13, 14, 15, 16, 17, 18, 19, 20, 21, 22, 23, 24, 25, 26, 27, 28, 29, 30, 31, 32, 33, 34, 35, 36, 37, 38, 39, 40, 41, 42, 43, 44, 45, 46, 47, 48, 49) represents tests performed using the same procedure, with one figure showing the real values and the other figure showing the average results.

The testing shows the effects of variations in item diversity and novelty on the results of the evaluation rules and how the new rules can be used to control these variations based on user behavior, leading to more precision and less variation of the results, which can help RSs suggest better evaluated items when searching for items with similar evaluations.

Table 2 summarizes all abbreviations used in the current section and in the experimental section below.

**Table 2:** Table of abbreviations.

Abbreviation	Description
div	Diversity
nov	Novelty
sugg	Suggestion
perc	Percentage
DB	Database/dataset
L_u_	Prediction list for user u
d (i,j)	Distance between items i and j (similarity value)
V_s_	Degree of variety of a selected item s
d (s,i)	Distance between selected item s and another suggested item i
Var(V_n_)_u_	Average degree of variety of n evaluated items for user u
N_s_	Degree of newness of a selected item s
Newn (N_m_)_u_	Average degree of newness of m evaluated items for user u
k	Number of suggested items
n	Number of entries in a set of V degree evaluations
m	Number of entries in a set of N degree evaluations

## 5 Experiments and solutions

The main goal is to provide a new evaluation model based on previous evaluation rules to improve the assessment of RS approaches by developing new concepts for evaluation related to some important RS characteristics (i.e., novelty and diversity).

**Figure 5:**
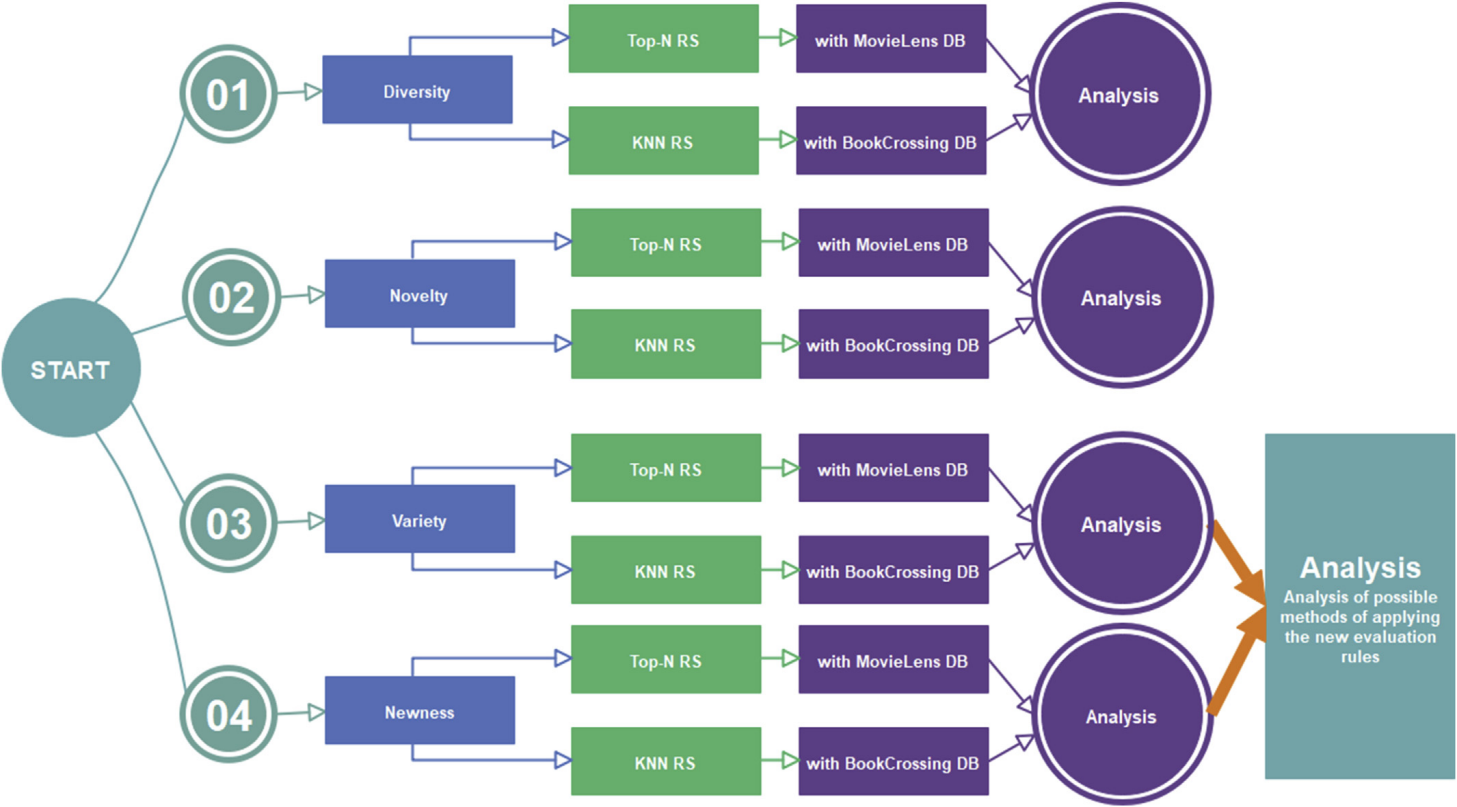
Testing methodology.

We test evaluation rules (Figure 5: experiments steps) based on both previous (diversity and novelty) and new (variety and newness) concepts using two CF-based RSs (a top-N RS and a KNN RS) on two officially available online databases (MovieLens and BookCrossing) [41]. The target of these experiments is not to compare the old and new evaluation rules; rather, the results illustrate the effects of an increasing percentage of unexpected suggestions on the outcomes of these rules, demonstrating the need for a “degree of control” and finding the appropriate evaluation ratio to achieve optimal levels of user attraction and satisfaction. In testing for diversity, novelty, variety, and newness, there is no change of RS algorithms, however, testing changes the final suggestions list delivered to the user, and the evaluations are based on this list.

All experiments (Figures 6, 7, 8, 9, 10, 11, 12, 13, 14, 15, 16, 17, 18, 19, 20, 21, 22, 23, 24, 25, 26, 27, 28, 29, 36, 37, 38, 39, 40, 41, 42, 43) that are not related to user selections are offline experiments. The experiments (Figures 30, 31, 32, 33, 34, 35, 44, 45, 46, 47, 48, 49) that are related to user selections are also offline experiments but with real-time calculation of the evaluation degrees of the selections and updating of the user profiles. The results would not show major changes in the case of online tests because the evaluations are related to selections of each user and her/his reactions to the decisions of the RS.

**Figure 6:**
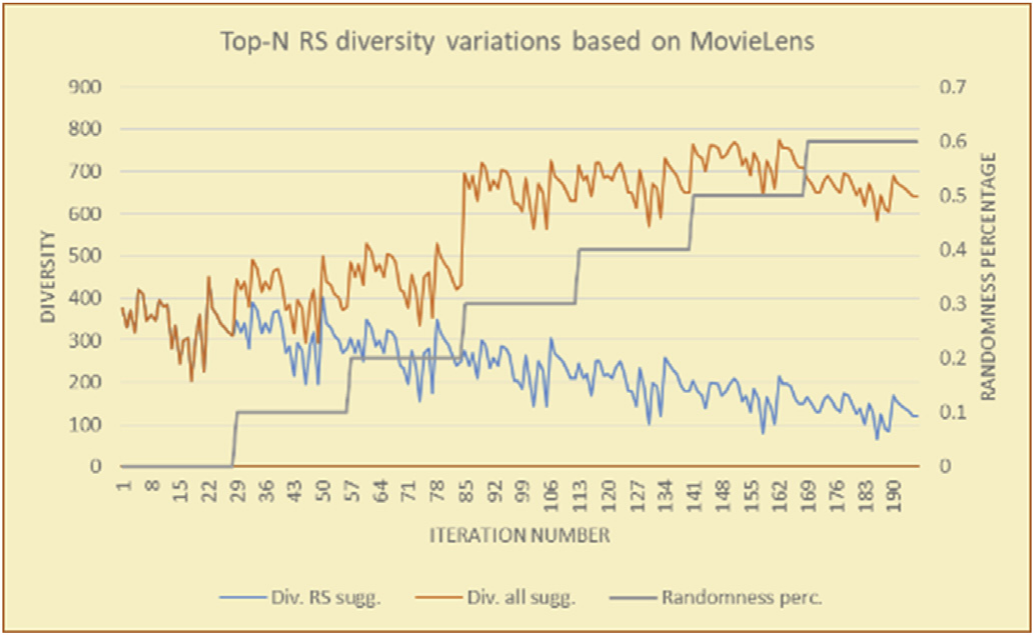
Effects on diversity of adding random suggestions for the top-N RS and the MovieLens DB.

**Figure 7:**
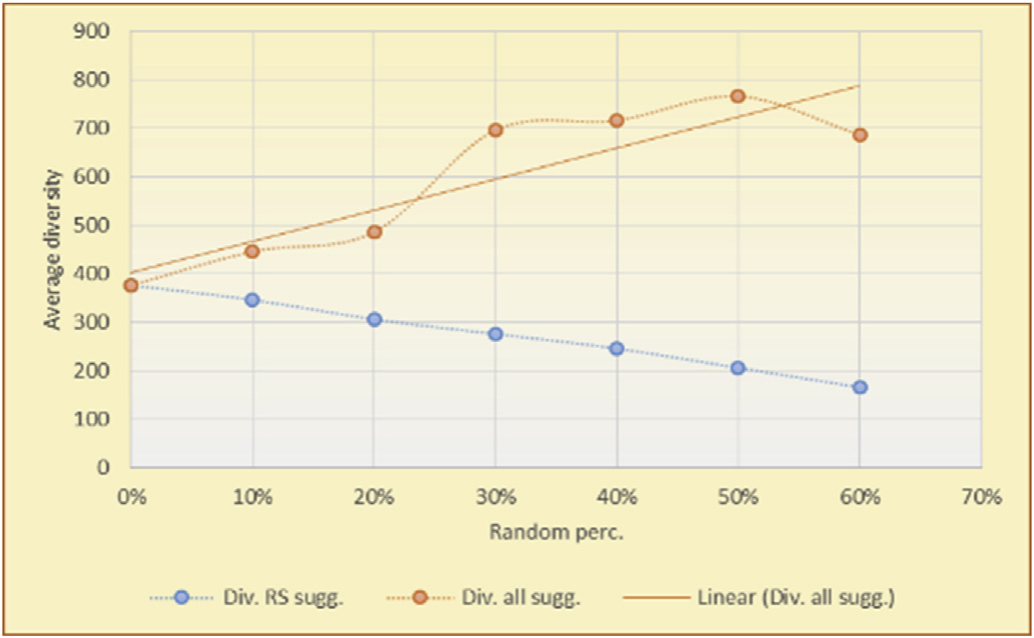
Averages of the results in Figure 6 for each randomness percentage.

**Figure 8:**
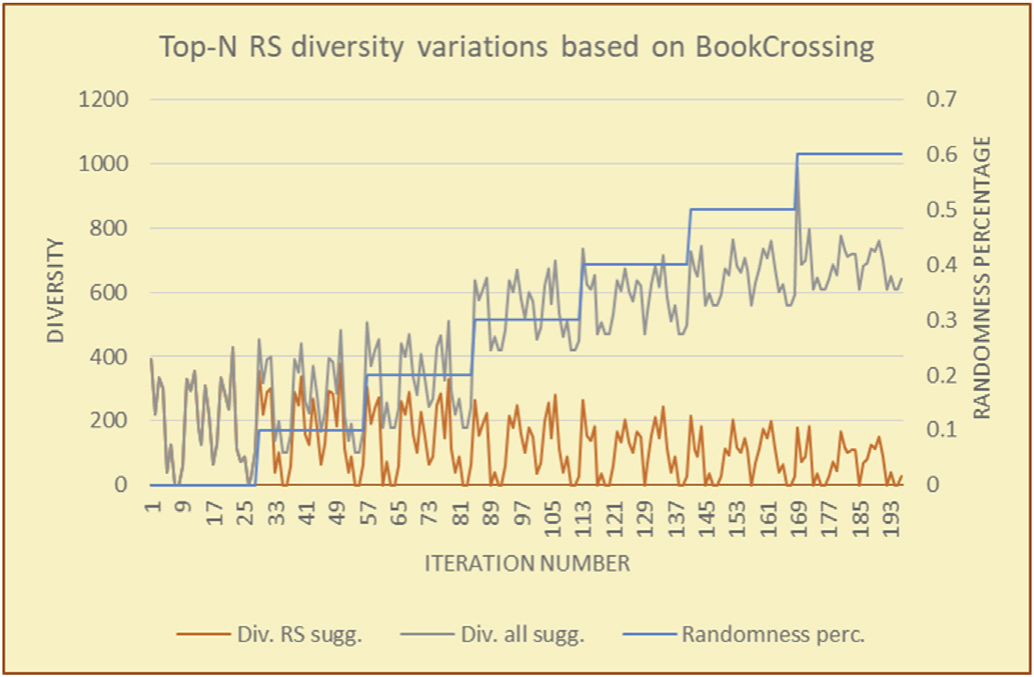
Effects on diversity of adding random suggestions for the top-N RS and the BookCrossing DB.

**Figure 9:**
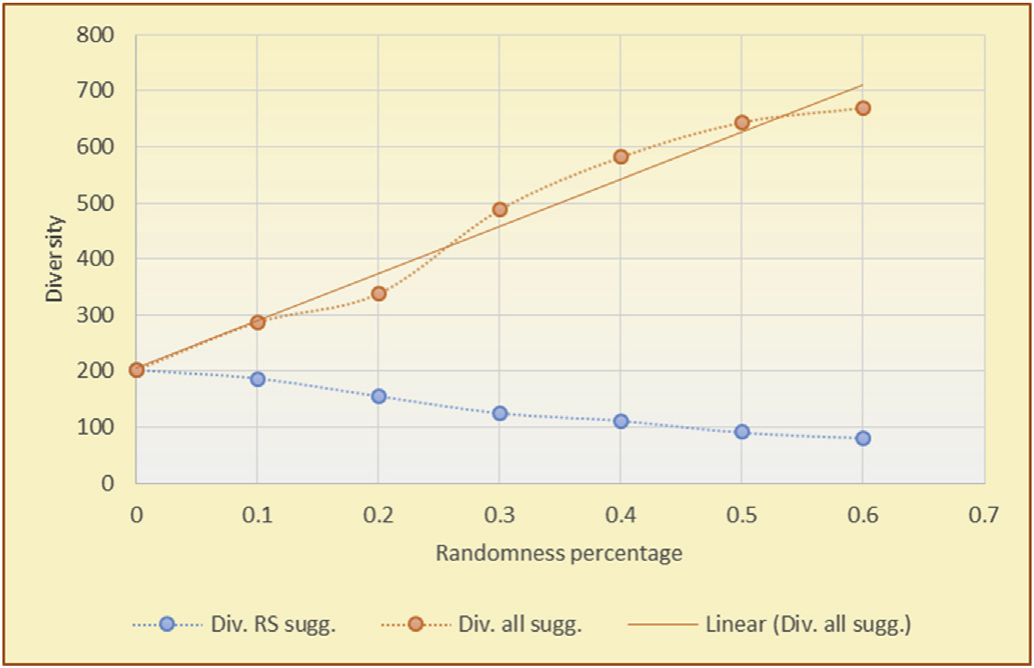
Averages of the results in Figure 8 for each randomness percentage.

**Figure 10:**
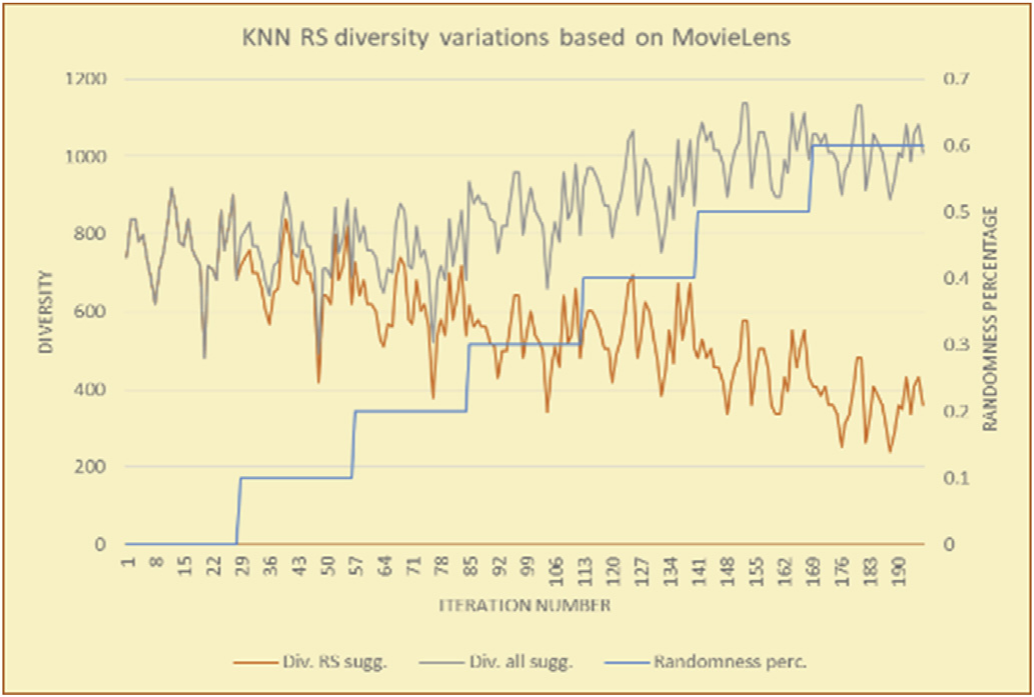
Effects on diversity of adding random suggestions for the KNN RS and the MovieLens DB.

**Figure 11:**
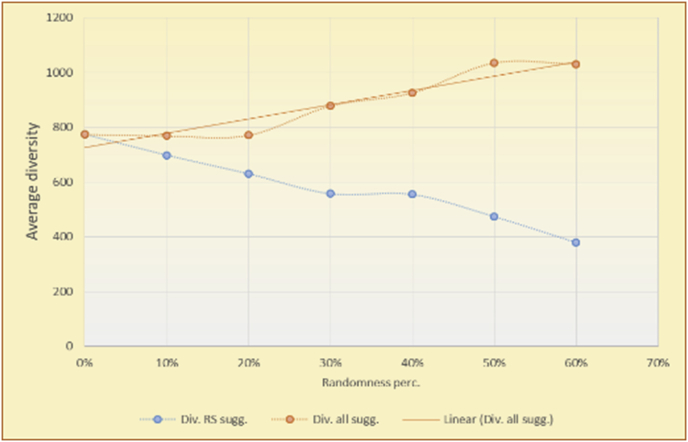
Averages of the results in Figure 10 for each randomness percentage.

**Figure 12:**
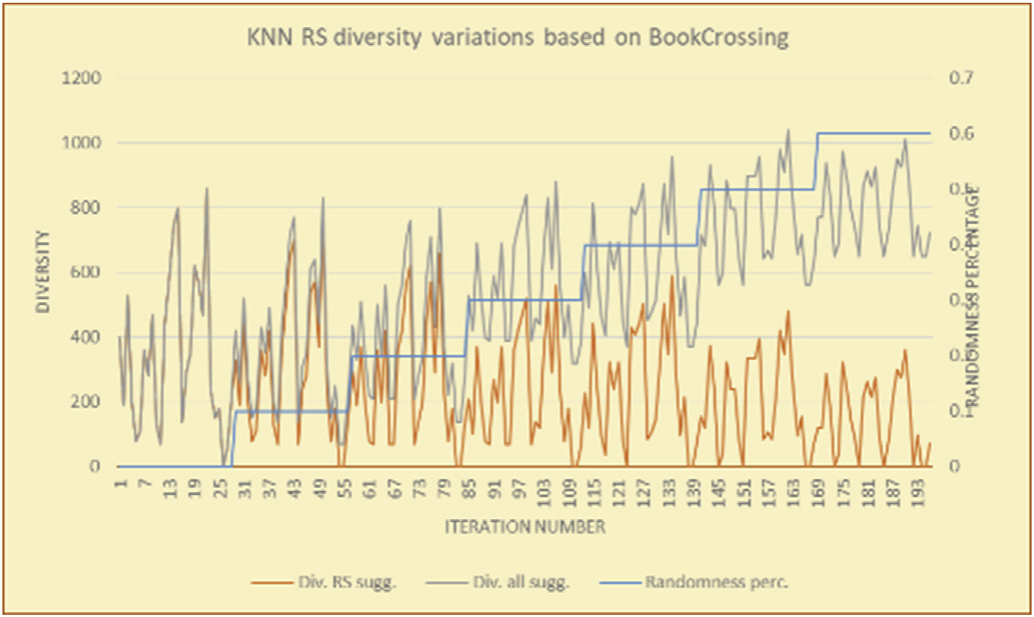
Effects on diversity of adding random suggestions for the KNN RS and the BookCrossing DB.

**Figure 13:**
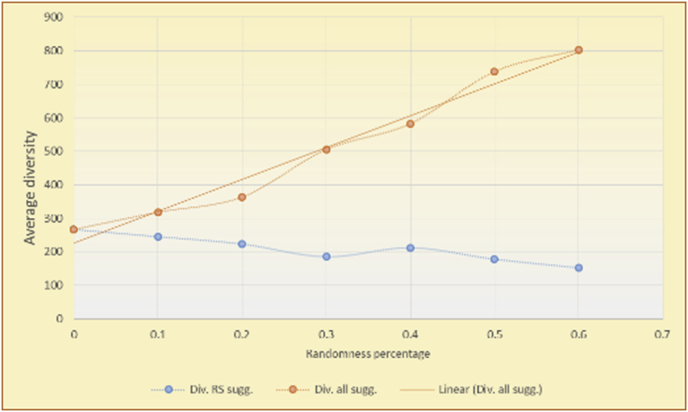
Averages of the results in Figure 12 for each randomness percentage.

**Figure 14:**
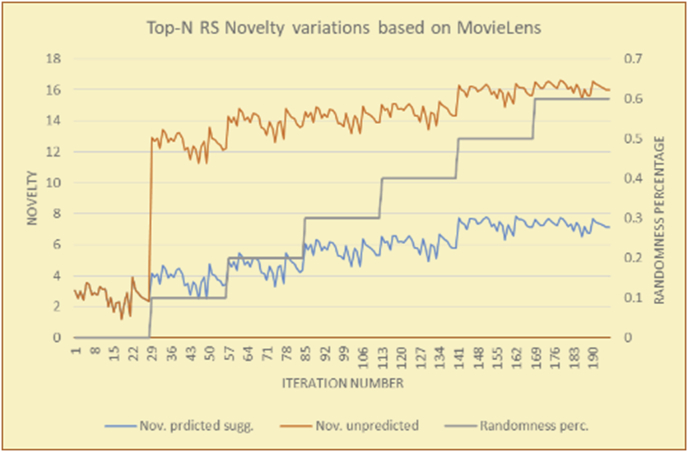
Effects on novelty of adding random suggestions for the top-N RS and the MovieLens DB.

**Figure 15:**
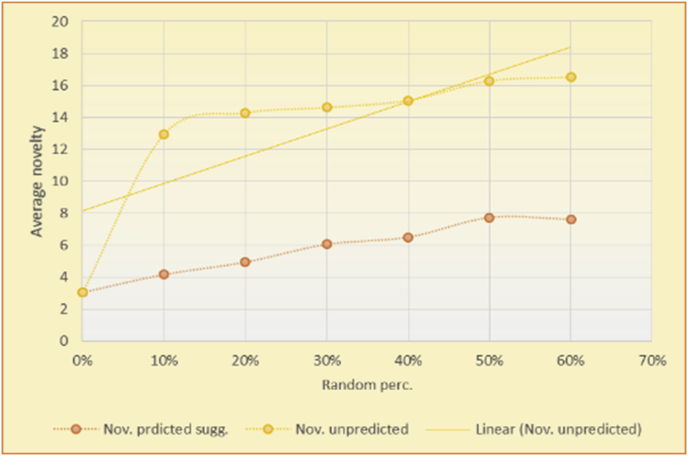
Averages of the results in Figure 14 for each randomness percentage.

**Figure 16:**
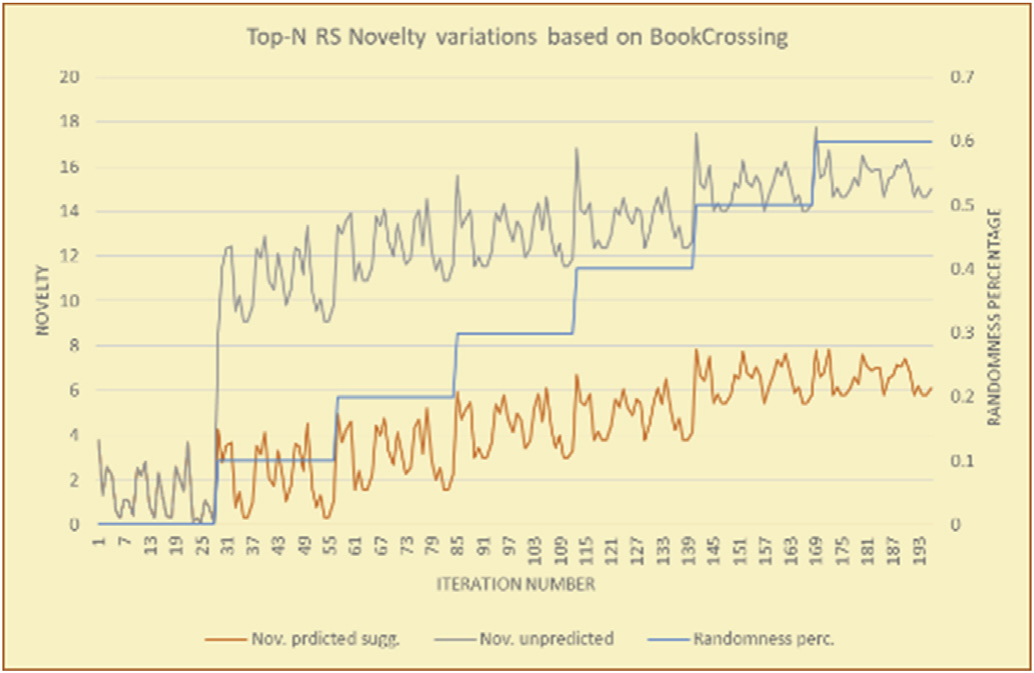
Effects on novelty of adding random suggestions for the top-N RS and the BookCrossing DB.

**Figure 17:**
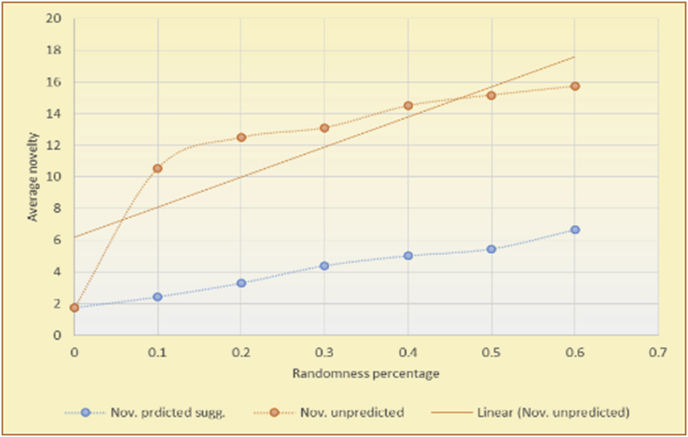
Averages of the results in Figure 16 for each randomness percentage.

**Figure 18:**
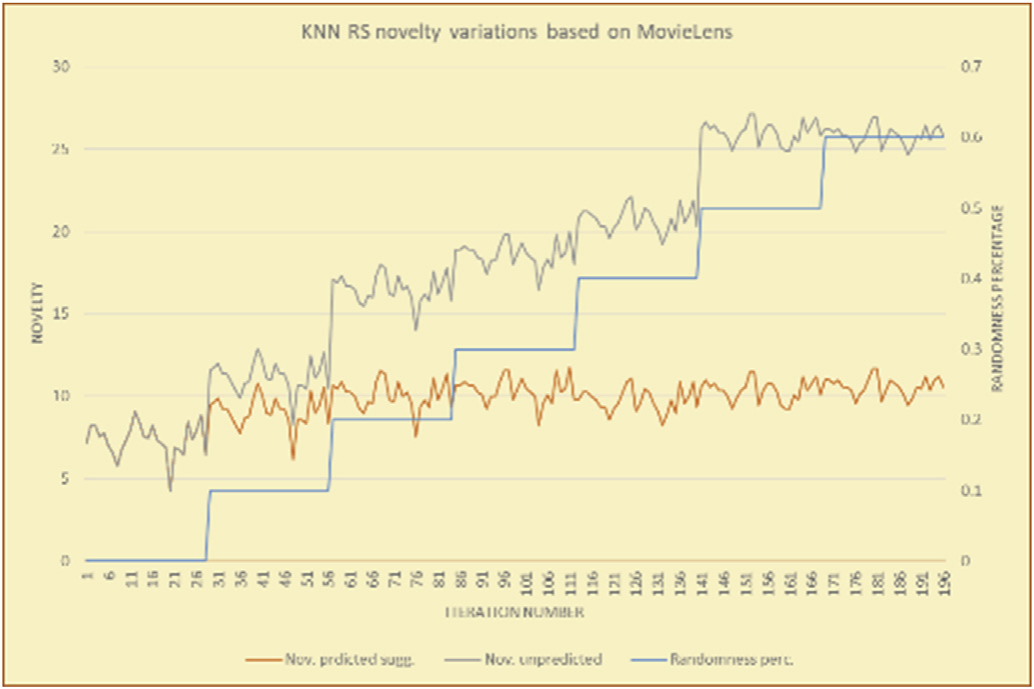
Effects on novelty of adding random suggestions for the KNN RS and the MovieLens DB.

**Figure 19:**
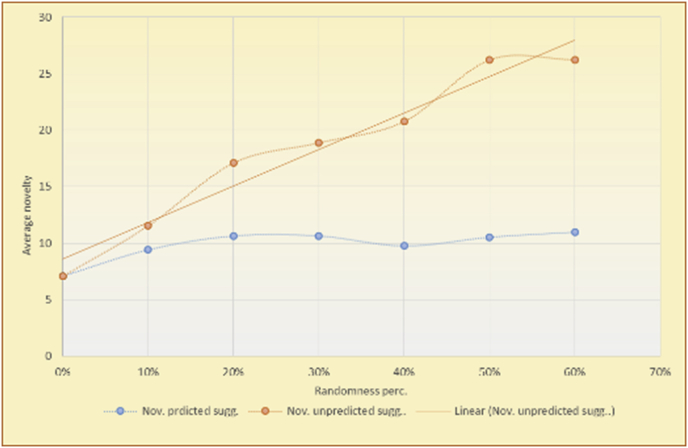
Averages of the results in Figure 18 for each randomness percentage.

**Figure 20:**
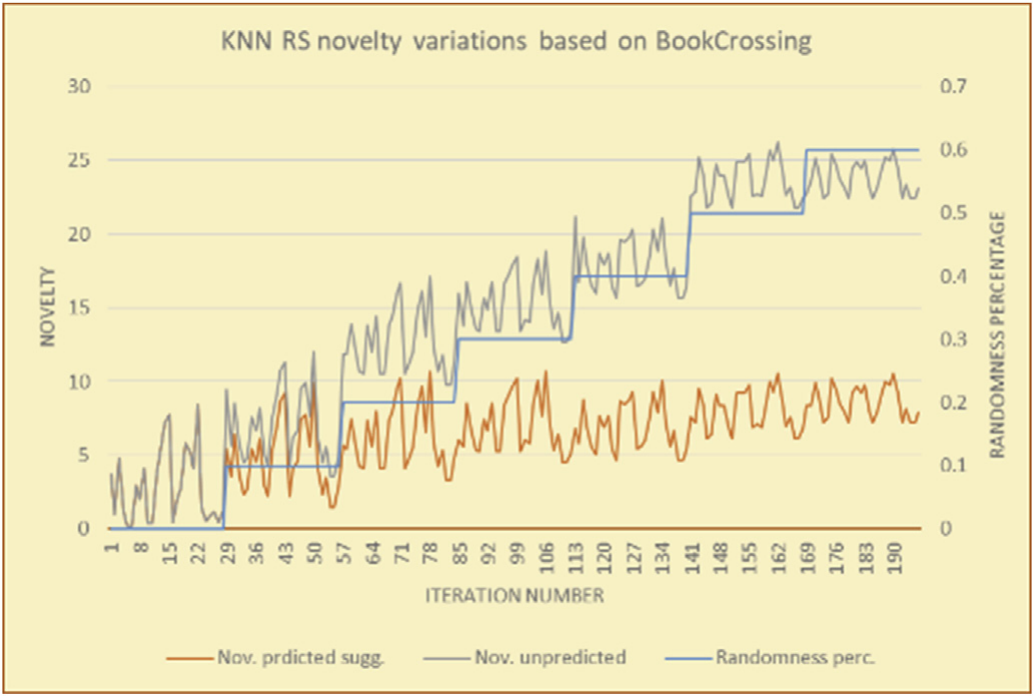
Effects on novelty of adding random suggestions for the KNN RS and the BookCrossing DB.

**Figure 21:**
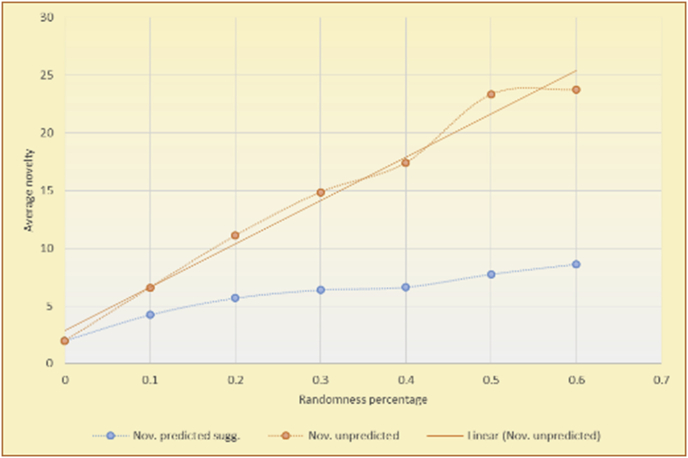
Averages of the results in Figure 20 for each randomness percentage.

**Figure 22:**
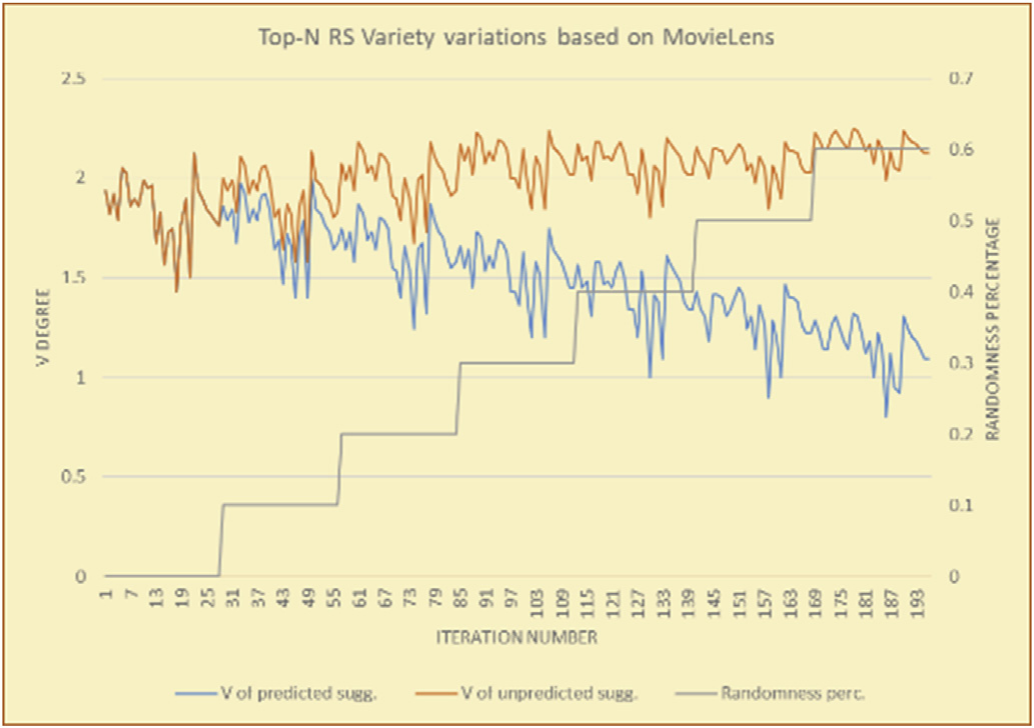
Degrees of variety for the top-N RS and the MovieLens DB.

**Figure 23:**
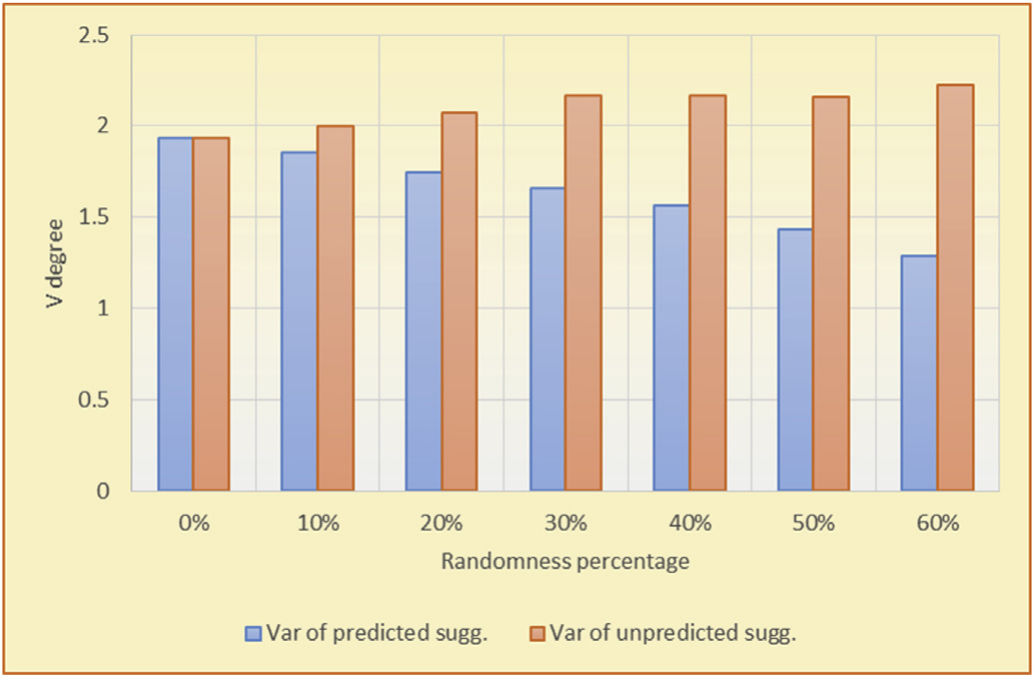
Averages of the results in Figure 22 for each randomness percentage.

**Figure 24:**
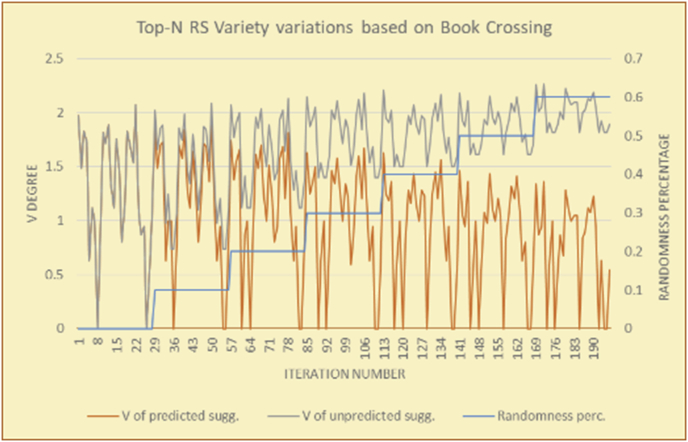
Degrees of variety for the top-N RS and the BookCrossing DB.

**Figure 25:**
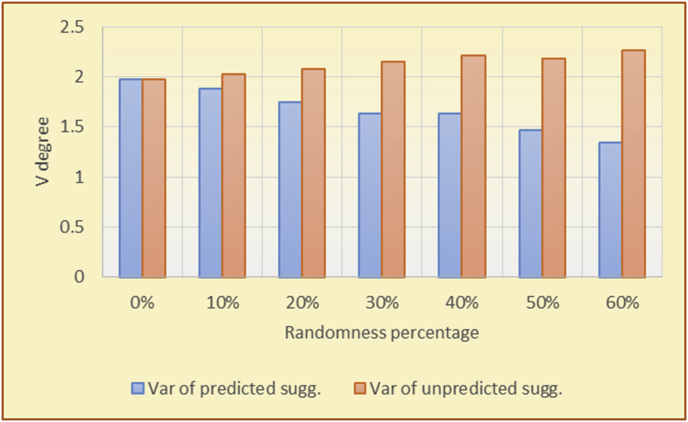
Averages of the results in Figure 24 for each randomness percentage.

**Figure 26:**
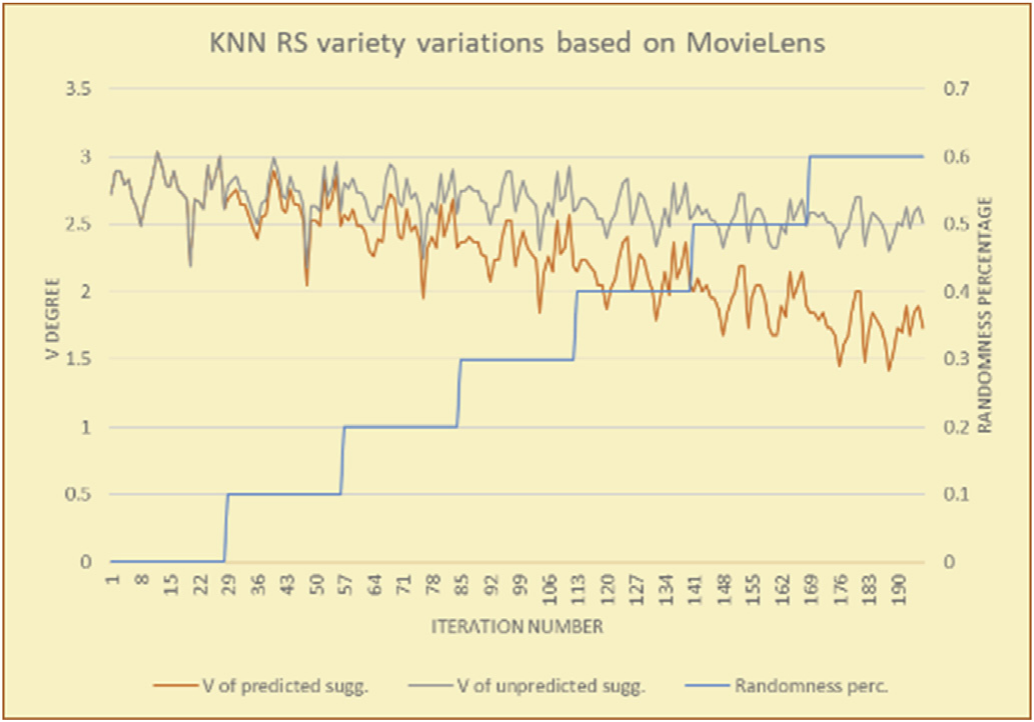
Degrees of variety for the KNN RS and the MovieLens DB.

**Figure 27:**
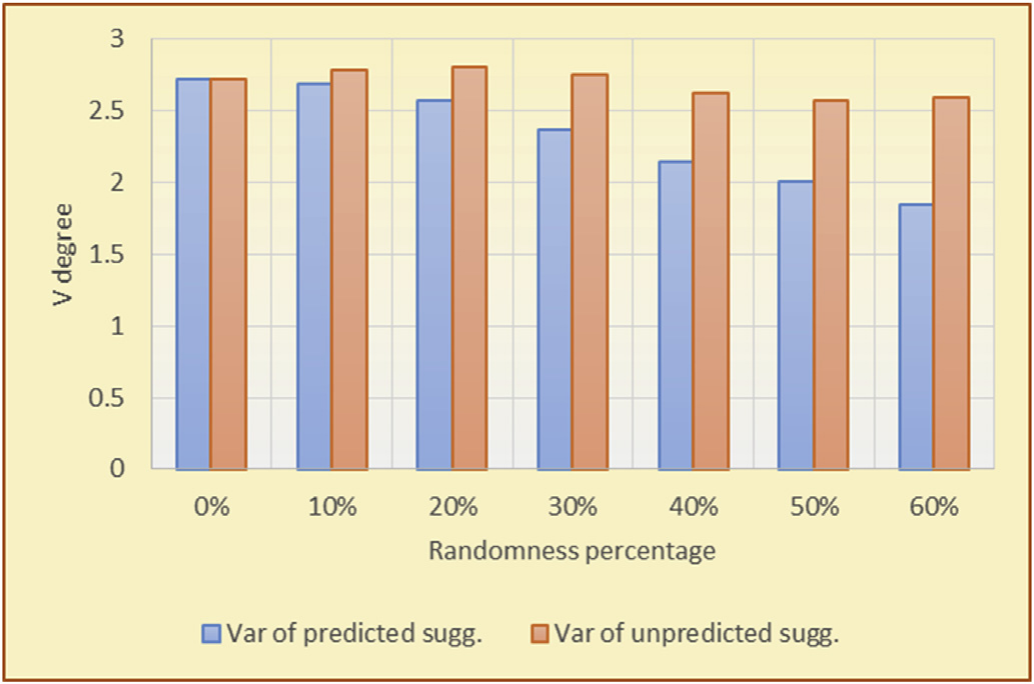
Averages of the results in Figure 26 for each randomness percentage.

**Figure 28:**
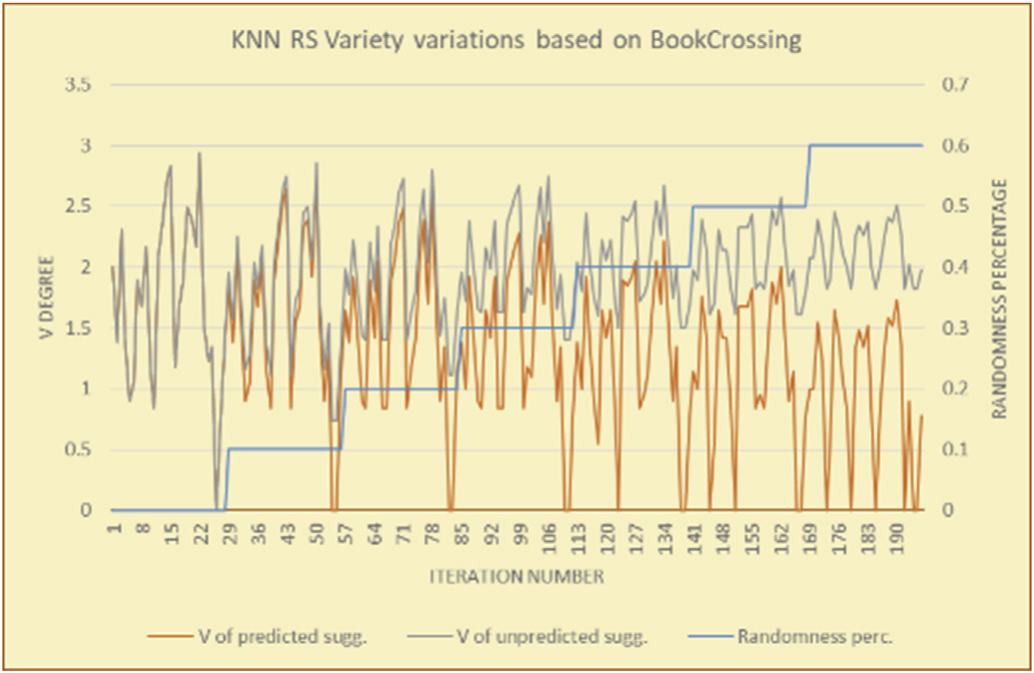
Degrees of variety for the KNN RS and the BookCrossing DB.

**Figure 29:**
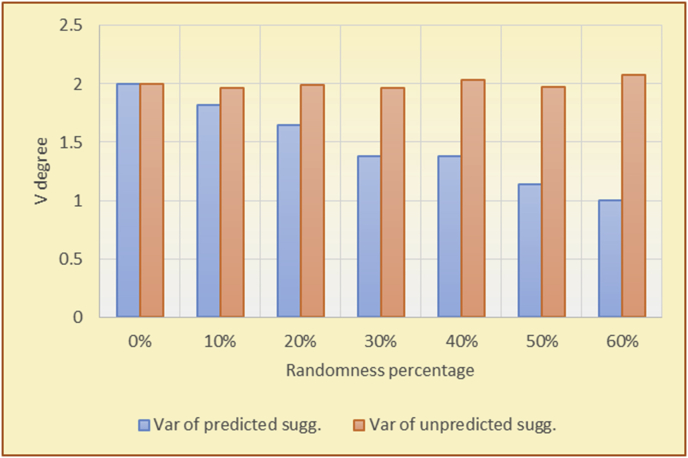
Averages of the results in Figure 28 for each randomness percentage.

**Figure 30:**
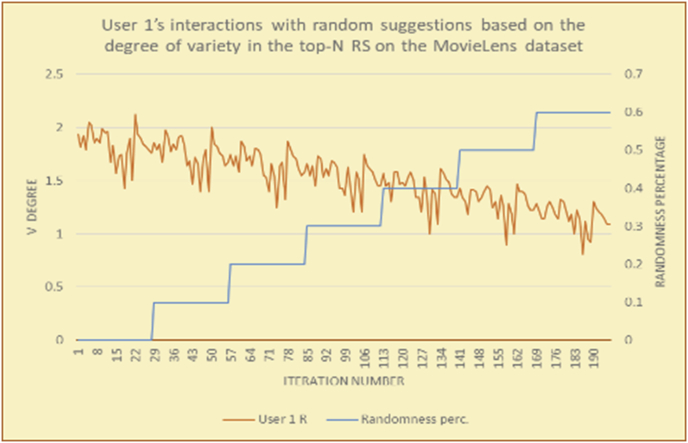
Degrees of variety for user 1.

**Figure 31:**
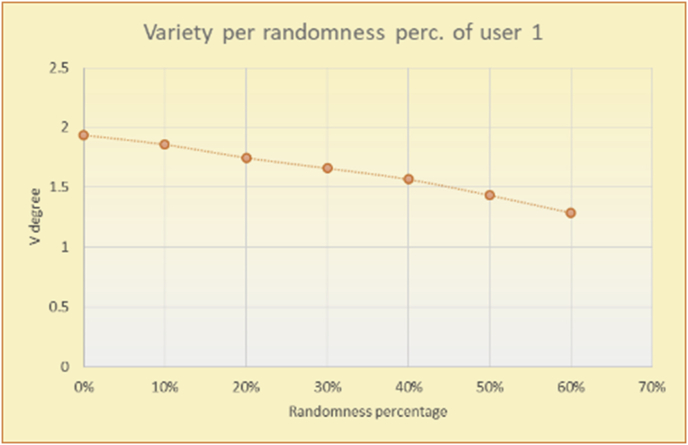
Average variety for user 1.

**Figure 32:**
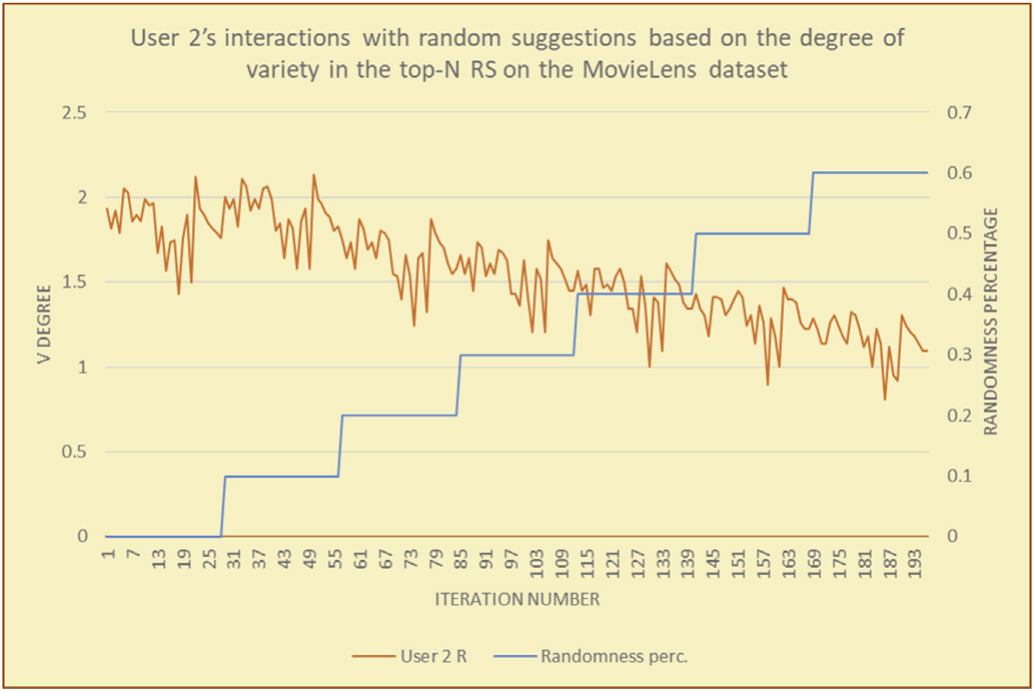
Degrees of variety for user 2.

**Figure 33:**
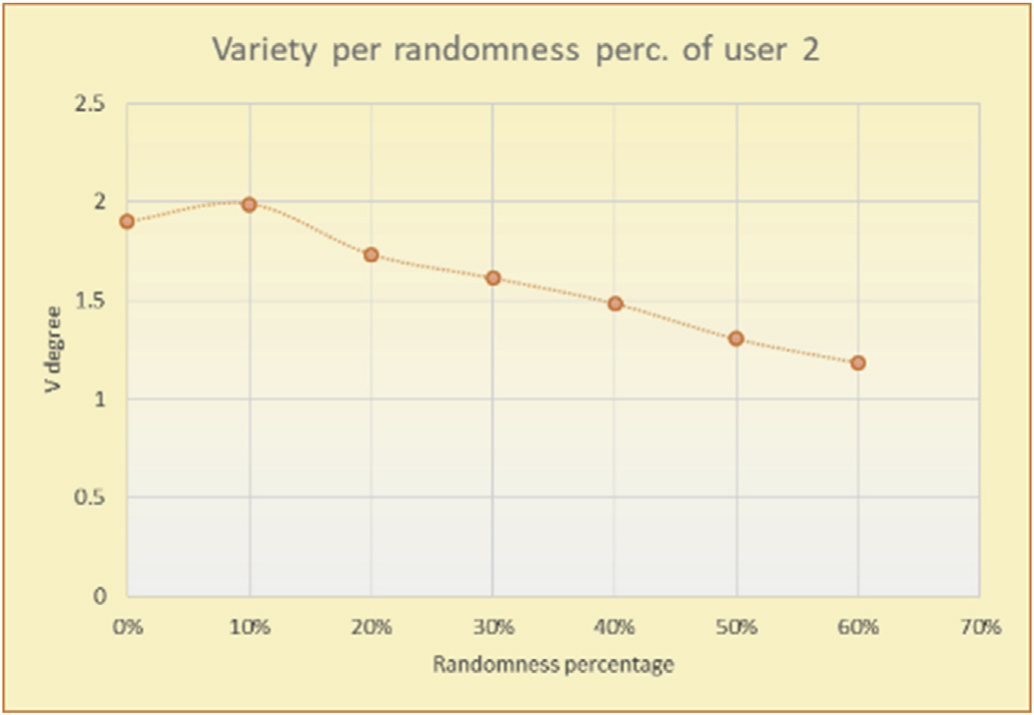
Average variety for user 2.

**Figure 34:**
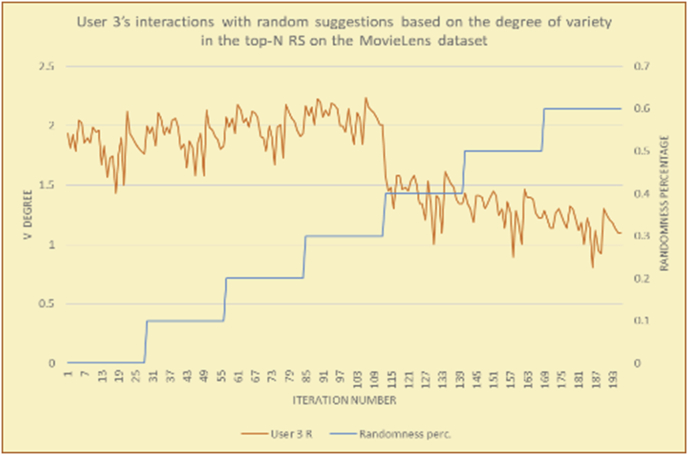
Degrees of variety for user 3.

**Figure 35:**
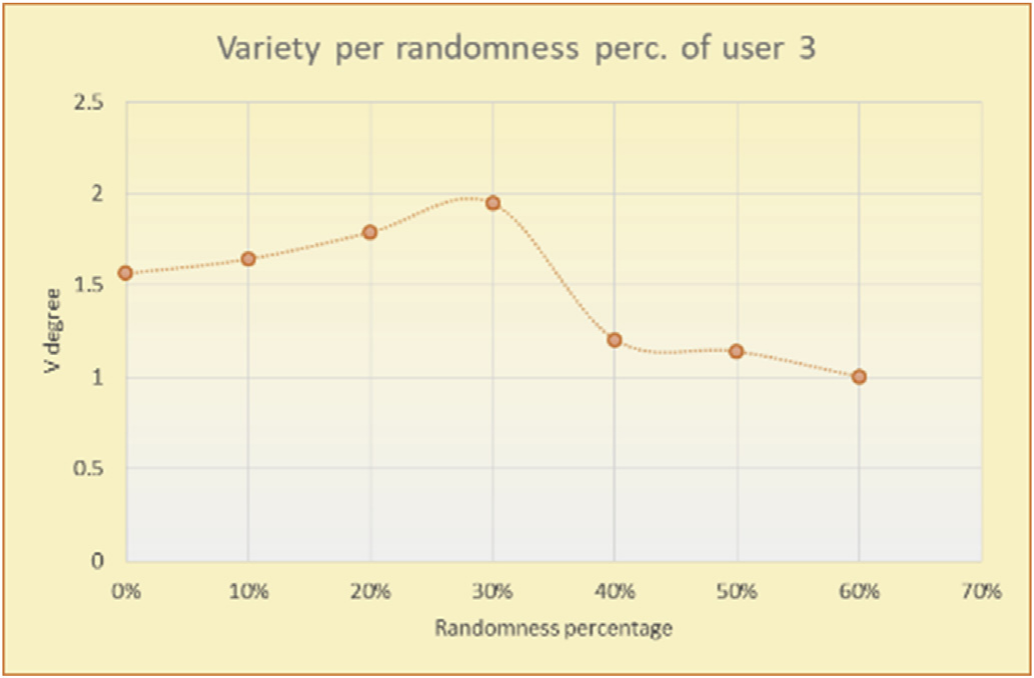
Average variety for user 3.

### 5.1 Diversity and novelty testing

It is hard to read test results without dividing them into phases, based on the percentage of random suggestions added in each phase, therefore all of the research's details tests (like Figures 6, 8, 10, 12) are presented in phases charts (like Figures 7, 9, 11, 13), and testing analysis will be based on the phases charts. Table 3 describes, by numbers, the comparison between random percentage phases and the different types of testing in order to show the average charts in numerical detailed explanation. As shown in Figures 6, 7, 8, 9, 10, 11, 12, 13 and Table 3, the diversity of the original RS suggestions decreases (367.14–151.42, 774.28 to 380.57, 201.42 to 80.71, and 267.14 to 152.57) as their percentages that are replaced by random items decreases, but the diversity of all final suggestions (generated by the system and at random) increases in an irregular manner. On the other hand, the diversity of all suggestions increases in an irregular manner (367.14–671.42, 774.28 to 1030, 201.42 to 669.28, and 267.14 to 802.57) with the increasing random percentage, due to the new random item diversification in the suggestion list. These findings show the high and irregular impact of random suggestions on diversity.

**Table 3:** Numerical comparison of diversity testing based on phases results.

Diversity testing	MovieLens DB				BooKCrossing DB			
	Top N RS		KNN RS		Top N RS		KNN RS	
Random percentage	Div. RS sugg.	Div. all sugg.	Div. RS sugg.	Div. all sugg.	Div. RS sugg.	Div. all sugg.	Div. RS sugg.	Div. all sugg.
0%	367.14	367.14	774.28	774.28	201.42	201.42	267.14	267.14
10%	337.14	437.14	698.57	768.57	186.42	286.42	245.71	318.57
20%	298.57	478.57	630.85	770.85	155	337.85	223.85	363.85
30%	260	680	558	878	125	487.85	185.57	505.57
40%	222.85	692.85	555.42	925.42	111.42	581.42	212.57	582.57
50%	184.28	744.28	474.85	1034.85	90.71	643.57	178.28	738.28
60%	151.42	671.42	380.57	1030.57	80.71	669.28	152.57	802.57

These tests aim to show the effect of replacing suggested items with new and more diverse items on the diversity of RS suggestions. The point also affects user acceptance of the suggestions list, which should be tested in order to provide the right modification of suggestions based on a measurement of user acceptance.

Table 4 describes a detailed comparison between random percentage phases, and between the different types of testing in order to show the novelty average charts in numerical explanation.

**Table 4:** Numerical comparison of novelty testing based on phases results.

Novelty testing	MovieLens DB				BooKCrossing DB			
	Top N RS		KNN RS		Top N RS		KNN RS	
Random percentage	Nov. predicted sugg.	Nov. unpredicted	Nov. predicted sugg.	Nov. unpredicted	Nov. predicted sugg.	Nov. all sugg.	Nov. predicted sugg.	Nov. all sugg.
0%	2.96	2.96	7.49	7.49	1.52	1.52	1.96	1.96
10%	4.07	12.85	9.2	11.31	2.4	10.54	4.22	6.6
20%	4.87	14.20	10.23	16.68	3.27	12.48	5.68	12.09
30%	5.88	14.44	10.55	18.77	4.38	13.1	6.39	14.87
40%	6.25	14.8	9.8	20.8	5.01	14.49	6.63	18.82
50%	7.49	16.04	10.5	26.17	5.45	15.16	6.76	23.33
60%	7.46	16.34	10.74	25.96	6.67	15.72	8.63	23.74

The novelty of the system suggestions remains low, with a slight increase as the percentage of random suggestions increases (2.96–7.46, 7.49 to 25.96, 1.52 to 6.67, and 1.96 to 8.63), which is expected as the number of system suggestions decreases (Figures 13, 14, 15, 16, 17, 18, 19, 20, 21). On the other hand, the novelty of all final suggestions (generated by the system and at random) also increases smoothly (2.96–16.34, 7.49 to 25.96, 1.52 to 15.72, and 1.96 to 23.74), but lies at a much higher value range than the novelty of the system suggestions alone because of the distances between these two types of suggestions. These findings illustrate the important impact of random suggestions on increasing RS novelty. Here we conclude the importance of controlling any possible risk of decreasing or increasing novelty and diversity using the replacement of a part of RS-suggested items. The research provides an evaluation value (variety) that represents the user acceptance of diversity, which helps RS control the suggestions' diversity level, and an evaluation value (newness) that represents the user acceptance level of novelty, which help RS control the suggestions’ novelty level, thereby solving the problem of how the RS can find the best satisfaction level of diversity and novelty.

### 5.2 Variety testing

For the testing of variety, two charts are presented for each test. The first chart shows the results for the degrees of variety of the RS suggestions and the random suggestions. The second chart shows the average variety values based on the results in the first chart. These experiments illustrate comparisons between the evaluations of the RS and random suggestions in the suggestion list as well as the effects of random (or unexpected) suggestions on the suggested items’ diversity.

The results for the degrees of variety (Figures 22, 23, 24, 25, 26, 27, 28, 29) in the two types of RSs (top-N and KNN) on the two datasets (MovieLens and BookCrossing) show differences between the variety of the selected random suggestions (new or diverse suggestions with respect to the predicted suggestions) and the variety of the selected RS-provided suggestions, which will later be used to explain the tendencies of the users themselves.

Table 5 describes by numbers the comparison between random percentage phases, and between the different types of testing in order to show a detailed presentation of the variety of average charts. The critical differences between V of a predicted and unpredicted suggestion of an RS with a DB type (example in Top N RS with MovieLens DB) show the diversification value of the item inside the suggestions list (example V = 1.83 of predicted sugg. and V = 1.97 of unpredicted sugg. at 10 percent random suggestions), and later the user-selected items’ variety values are saved in her/his profile, which will be used by the RS to indicate the user acceptance level of diversity inside the suggestions list.

**Table 5:** Numerical comparison of variety testing based on phases results.

Variety testing	MovieLens DB				BooKCrossing DB			
	Top N RS		KNN RS		Top N RS		KNN RS	
Random percentage	V of predicted sugg.	V of unpredicted sugg.	V of predicted sugg.	V of unpredicted sugg.	V of predicted sugg.	V of unpredicted sugg.	V of predicted sugg.	V of unpredicted sugg.
0%	1.91	1.91	2.78	2.78	1.39	1.39	1.56	1.56
10%	1.83	1.97	2.64	2.74	1.34	1.48	1.51	1.69
20%	1.72	2.05	2.49	2.73	1.17	1.62	1.42	1.83
30%	1.6	2.13	2.34	2.72	1.05	1.76	1.29	1.92
40%	1.49	2.11	2.15	2.62	1.001	1.81	1.25	1.98
50%	1.35	2.1	1.98	2.56	0.91	1.86	1.07	2.003
60%	1.22	2.19	1.77	2.54	0.87	2.01	0.99	2.12

To illustrate how the variety rule helps in discovering variety-driven behaviors from users' activities, we present the variety results for the user selections from the suggestion lists generated by the top-N RS using the MovieLens DB for three users, chosen from a set of offline selections for 25 users; the experimental results for the other users are similar to those for the three users presented here and do not provide any additional information. User 1 (Figures 30 and 31) is uninterested in the random suggestions in all of his/her experiments, as seen from the fact that all of his/her results are equal to the degrees of variety of the items predicted by the RS. User 2 (Figures 32 and 33) is interested in random suggestions only up to a level of 10%, implying that the optimal percentage of unexpected items to be added to the suggestion list for user 2 is 10%. By comparison, user 3 (Figures 34 and 35) shows more responsiveness to a higher diversity of items, with the average variety increasing until 30% of the recommendation list consists of random suggestions. Accordingly, the variety degree or variety responsiveness percentage can be added to the profile of each user to indicate the optimal randomness percentage for the highest level of user satisfaction and utility based on each user's own variety behavior.

RS will continue to work and generate with the previous efficiency level, but with a variety value system it will know the user's possible percentage of replacing RS suggestions by more diverse ones without decreasing user satisfaction, but with a good opportunity to gain more satisfaction by pushing user curiosity to choose a newly added item and spend more time using the RS.

### 5.3 Newness testing

For the testing of newness, as in the case of variety, two charts are presented for each test. The first chart shows the results for the degrees of newness of the RS suggestions and the random suggestions, and the second chart shows the average newness values based on the results in the first chart. These experiments illustrate comparisons between the evaluations of the RS and random suggestions in the suggestions list and reveal the variations in user behavior (a user's degree of acceptance for newness) seen when testing with the new rules. The results show the effects of random (or unexpected) suggestions on the novelty of the suggested items.

**Figure 36:**
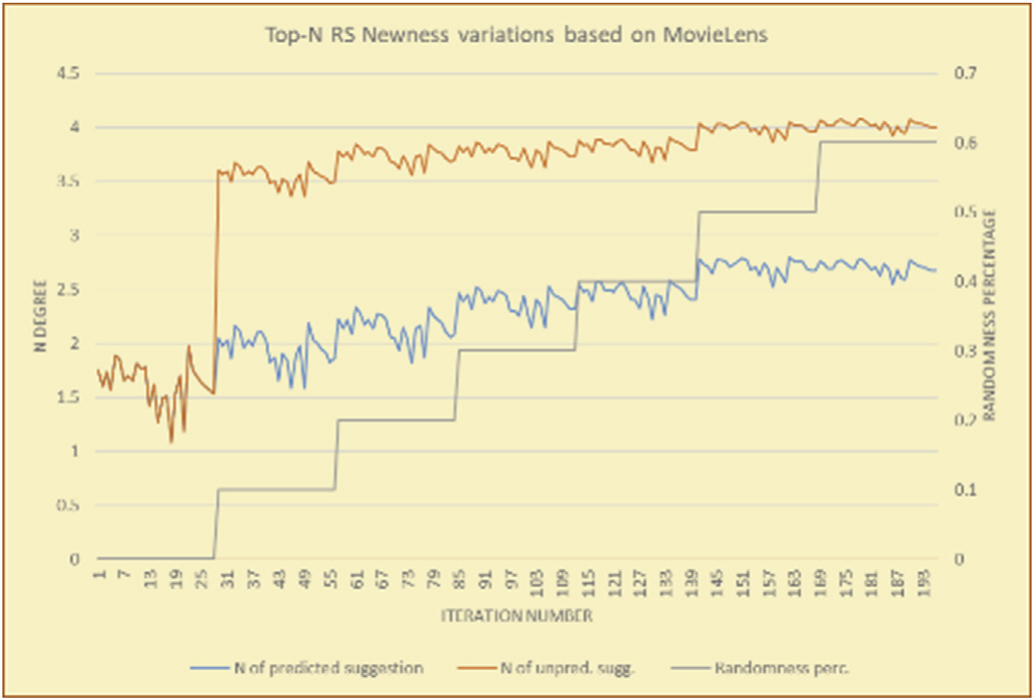
Degrees of newness for the top-N RS and the MovieLens DB.

**Figure 37:**
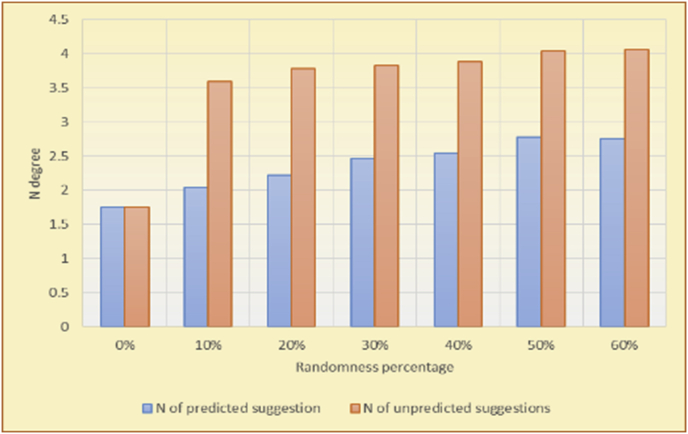
Averages of the results in Figure 36 for each randomness percentage.

**Figure 38:**
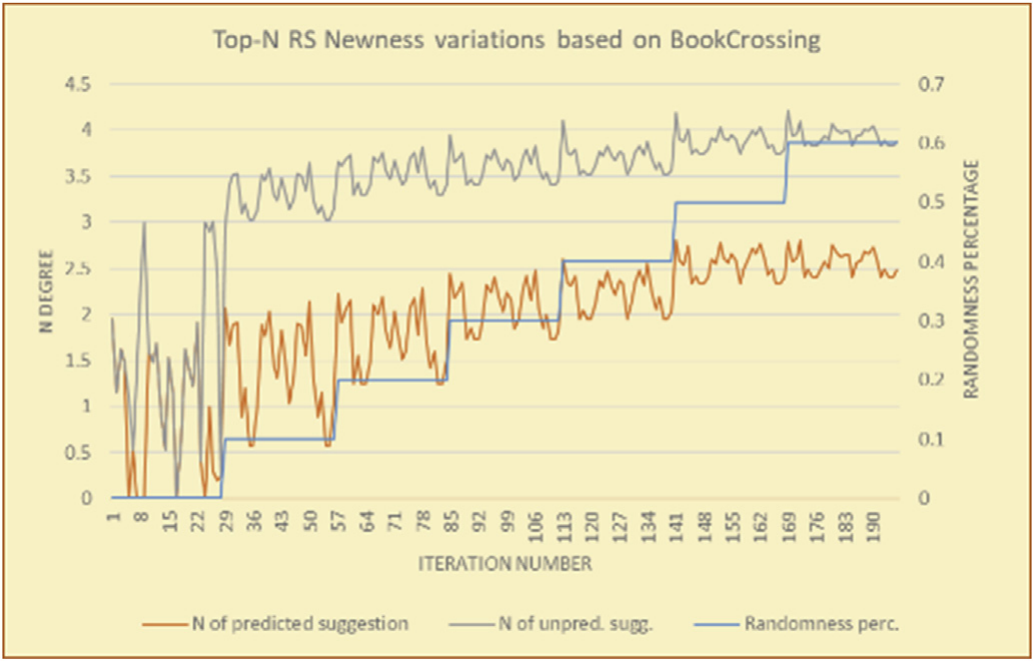
Degrees of newness for the top-N RS and the BookCrossing DB.

**Figure 39:**
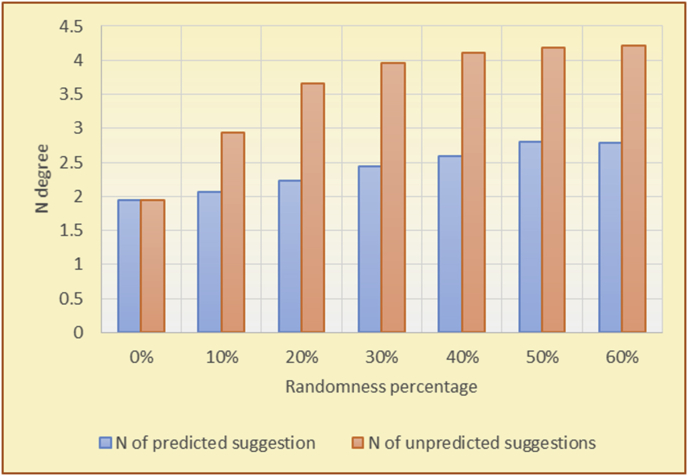
Averages of the results in Figure 38 for each randomness percentage.

**Figure 40:**
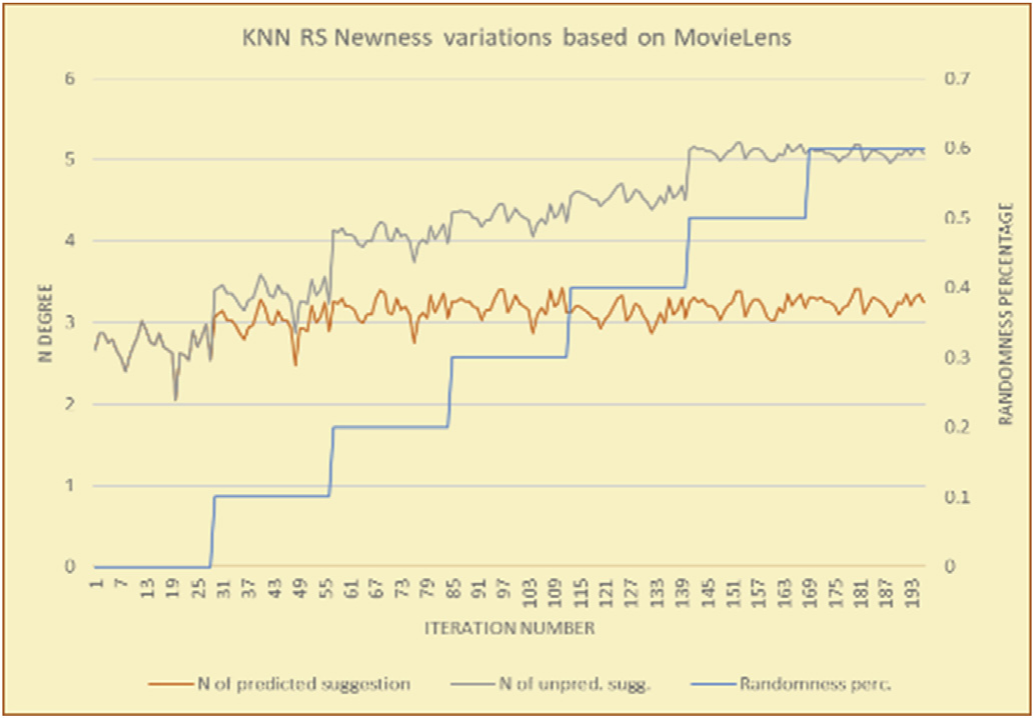
Degrees of newness for the KNN RS and the MovieLens DB.

**Figure 41:**
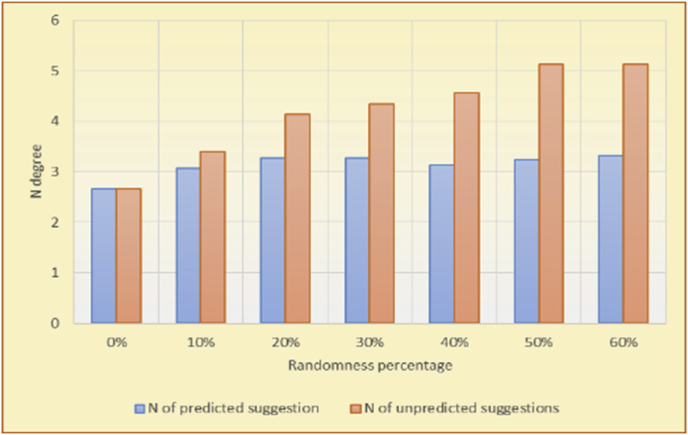
Averages of the results in Figure 40 for each randomness percentage.

**Figure 42:**
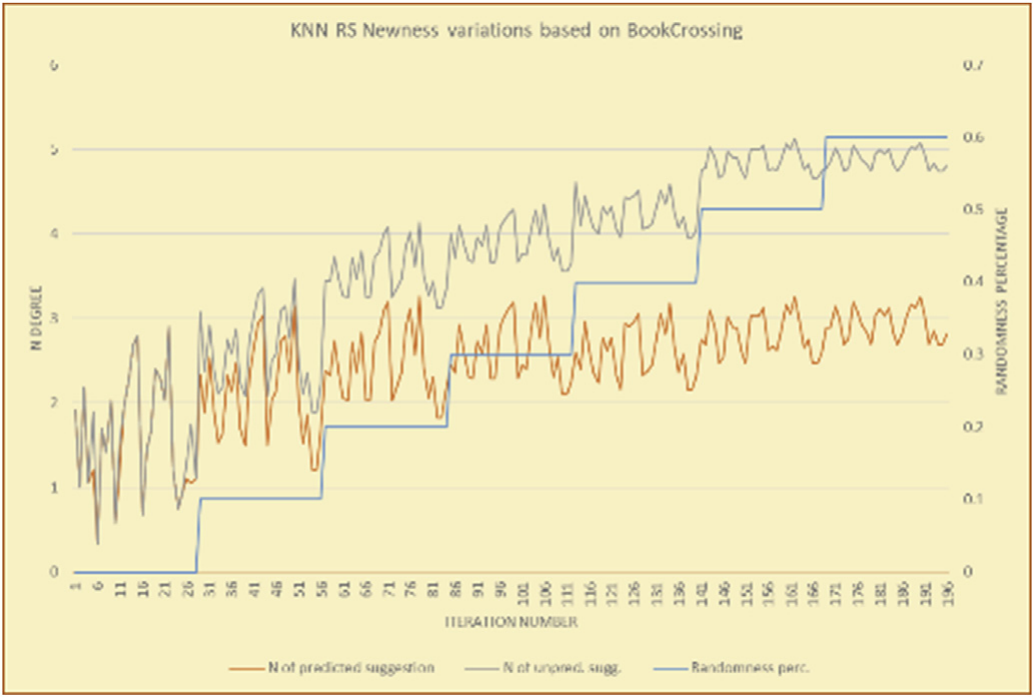
Degrees of newness for the KNN RS and the BookCrossing DB.

**Figure 43:**
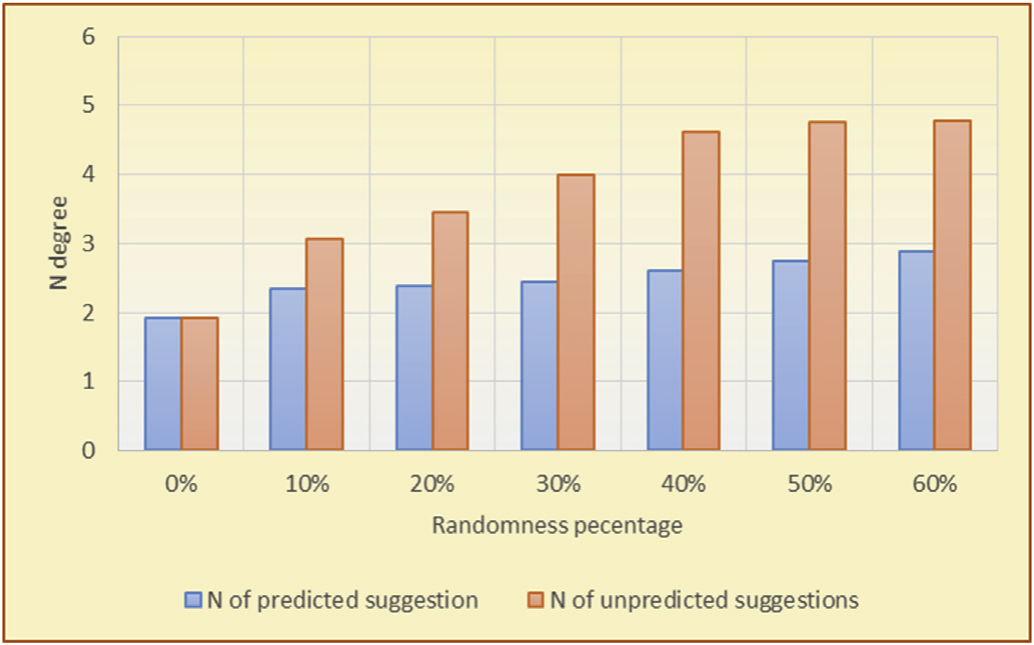
Averages of the results in Figure 42 for each randomness percentage.

The results for the degrees of newness (Figures 36, 37, 38, 39, 40, 41, 42, 43) in the two types of RSs (top-N and KNN) on the two datasets (MovieLens and BookCrossing) reveal the differences between the newness of the selected random suggestions and the newness of the selected RS-predicted suggestions (with respect to the predicted suggestions), which reflect the tendencies of the users themselves based on their selections.

Table 6 describes numerally the comparison between random percentage phases, and between the different types of testing in order to show a detailed presentation of the novelty average charts. The critical differences between N of a predicted and unpredicted suggestion of a RS with a DB type (example in Top N RS with MovieLens DB) show the novelty value of the item inside the suggestions list (example N = 2.01 of predicted sugg. and N = 3.58 of unpredicted sugg. at 10 percent random suggestions), so later the user-selected items’ newness values are saved in her/his profile, which will be used by the RS to indicate the user acceptance level of novelty inside the suggestions list.

**Table 6:** Numerical comparison of newness testing based on phases results.

Newness testing	MovieLens DB				BooKCrossing DB			
	Top N RS		KNN RS		Top N RS		KNN RS	
Random percentage	N of predicted sugg.	N of unpredicted sugg.	N of predicted sugg.	N of unpredicted sugg.	N of predicted sugg.	N of unpredicted sugg.	N of predicted sugg.	N of unpredicted sugg.
0%	1.71	1.71	2.73	2.73	0.96	1.3	1.34	1.44
10%	2.01	3.58	3.03	3.36	1.45	3.24	2.02	2.54
20%	2.2	3.76	3.19	4.08	1.76	3.52	2.36	3.47
30%	2.42	3.8	3.24	4.33	2.07	3.61	2.51	3.85
40%	2.5	3.84	3.13	4.56	2.22	3.7	2.56	4.25
50%	2.73	4.005	3.24	5.11	2.53	3.89	2.77	4.82
60%	2.73	4.04	3.27	5.09	2.57	3.96	2.93	4.87

**Figure 44:**
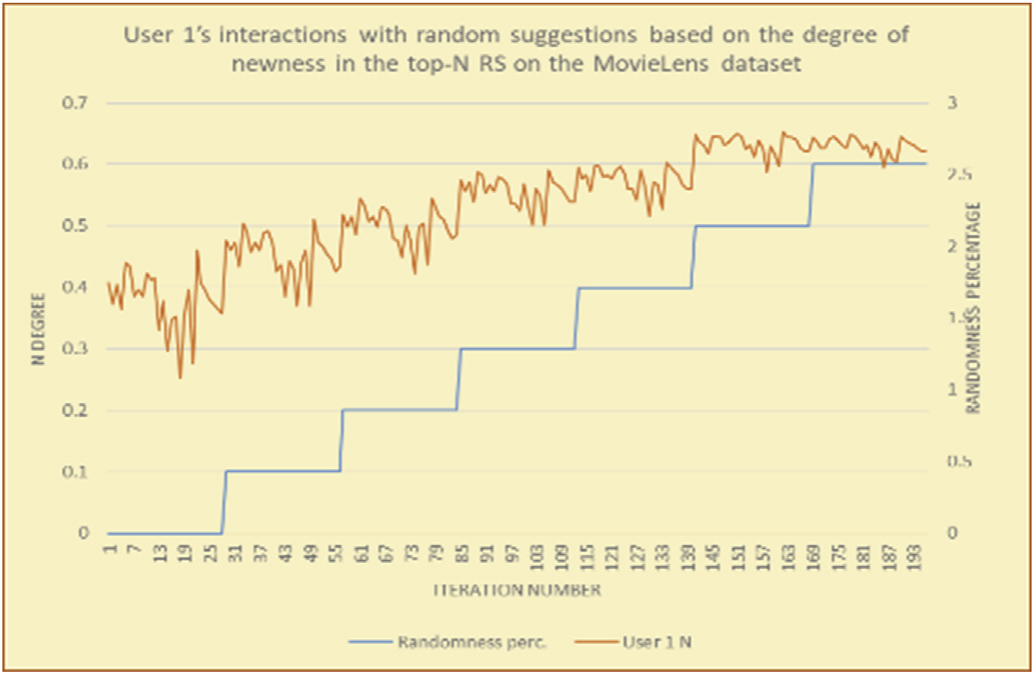
Degrees of newness for user 1.

**Figure 45:**
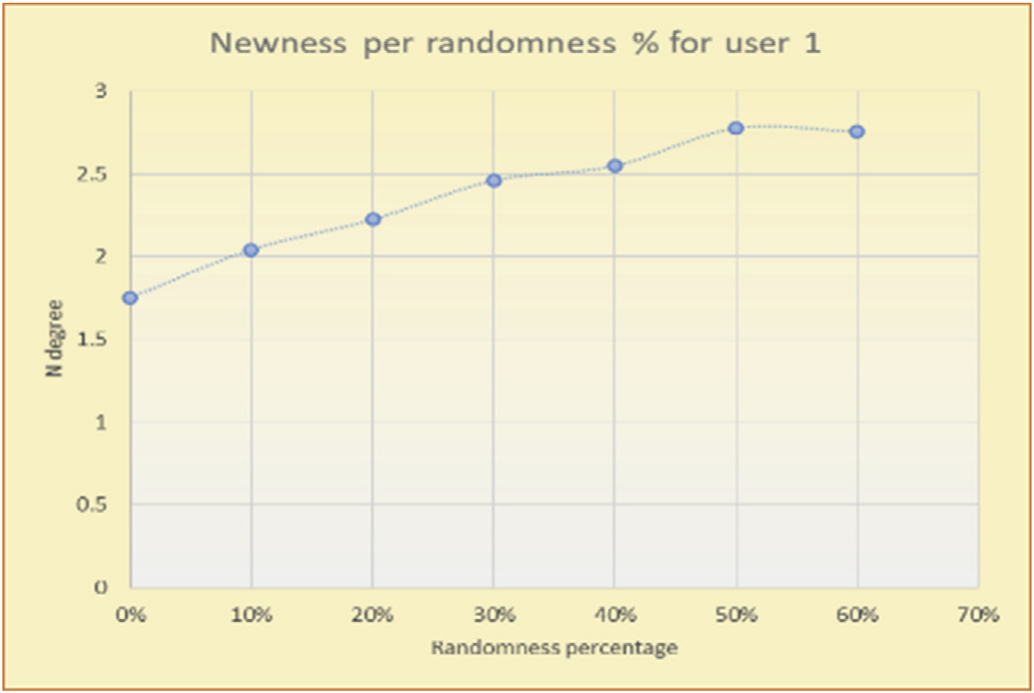
Average newness for user 1.

**Figure 46:**
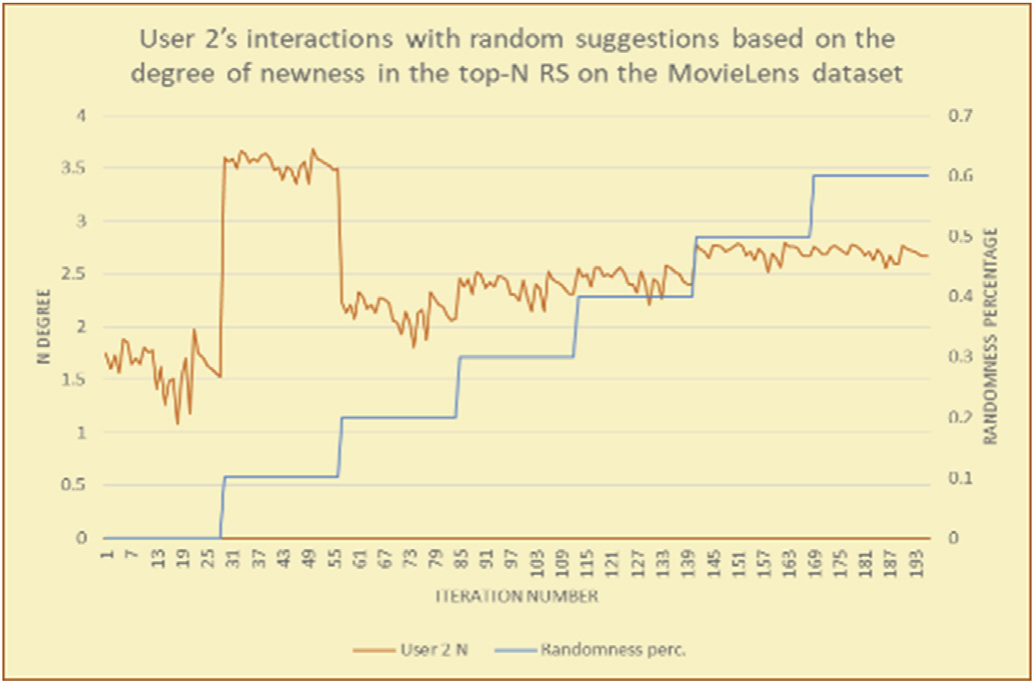
Degrees of newness for user 2.

**Figure 47:**
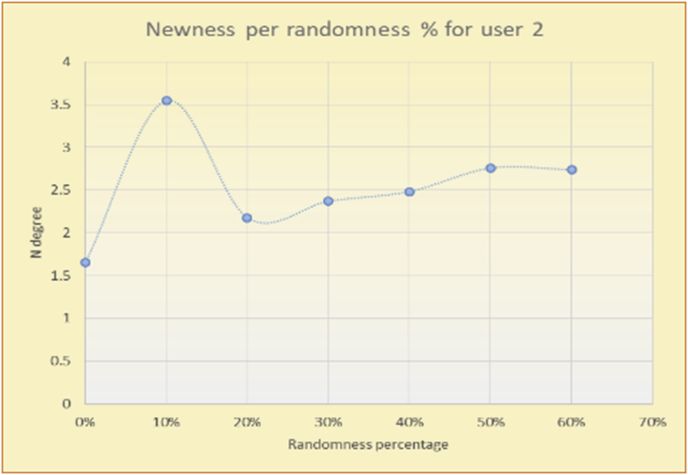
Average newness for user 2.

**Figure 48:**
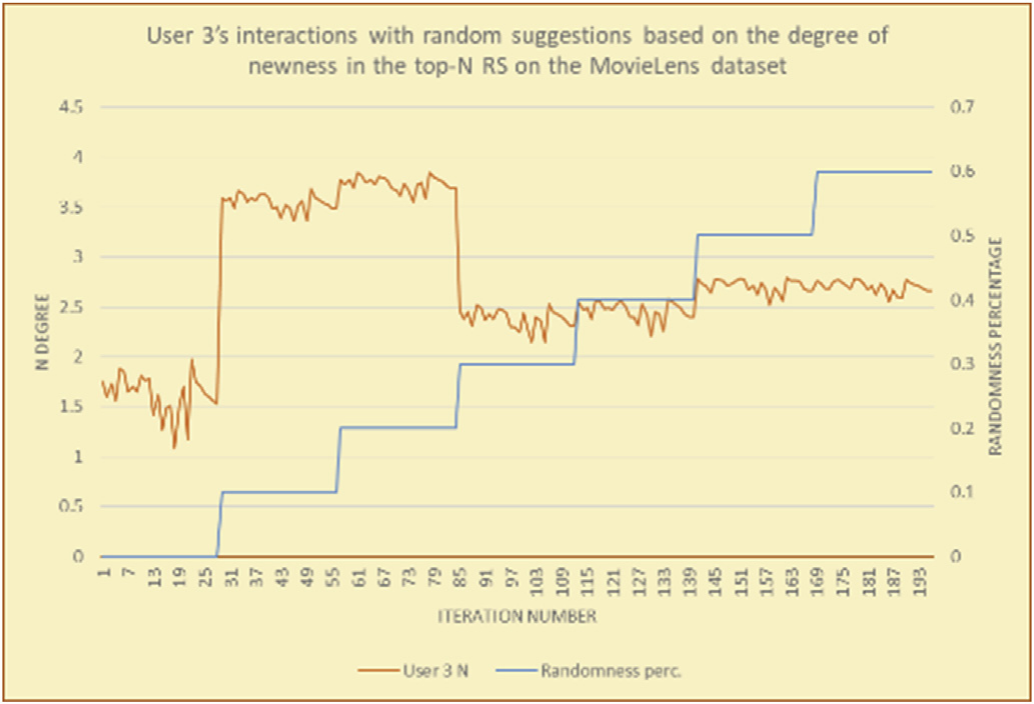
Degrees of newness for user 3.

**Figure 49:**
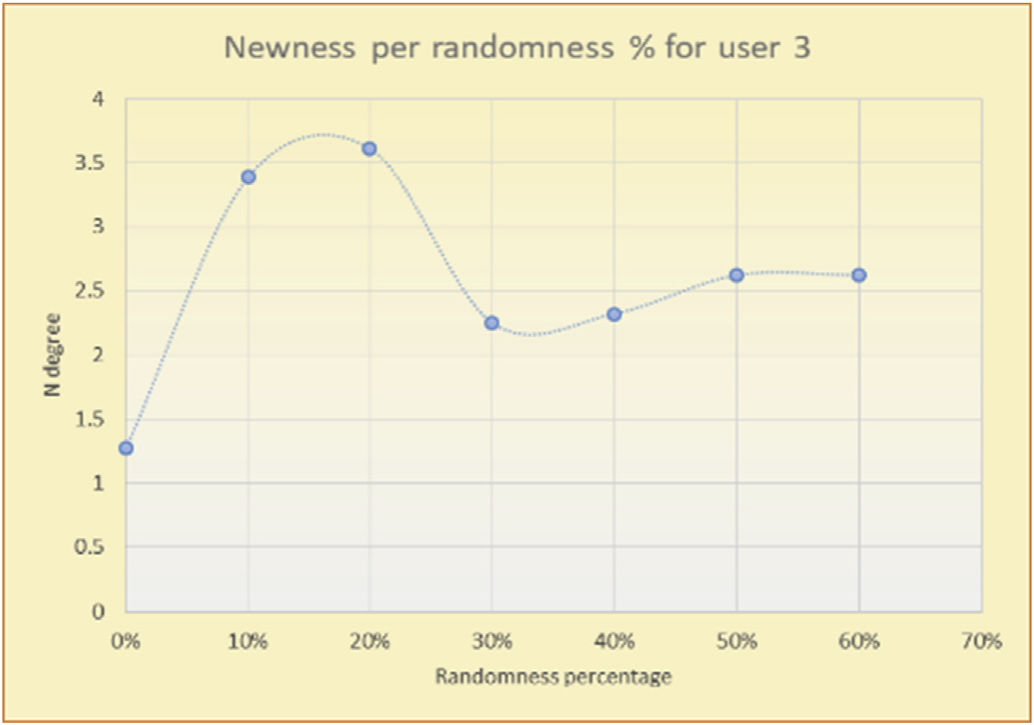
Average newness for user 3.

To illustrate how the newness rule helps in discovering the newness-oriented behaviors of individual users, we present the newness results for the user selections from the suggestion lists generated by the top-N RS using the MovieLens DB for three users, chosen from a set of offline selections for 25 users. User 1 (Figures 44 and 45) is uninterested in the random suggestions in all of his/her experiments, as seen from the fact that all of his/her results are equal to the degrees of newness of the items predicted by the RS. User 2 (Figures 46 and 47) is interested in random suggestions only up to a level of 10%, implying that the optimal percentage of unexpected items to be added to the suggestion list for user 2 is 10%. By comparison, user 3 (Figures 48 and 49) shows more responsiveness to new items, with the average newness increasing when up to 20% of the recommendation lists consists of added random suggestions. Accordingly, the newness degree or newness responsiveness percentage can be added to each user's profile to indicate the optimal randomness percentage for the highest level of user satisfaction and utility based on his/her newness behavior.

As for the newness rule, RS will continue to work and generate with its previous efficiency level, but with a newness value system will know the user's possible percentage of replacing RS suggestions with new or unexpected ones without decreasing user satisfaction, but with a good opportunity to gain more satisfaction by pushing user curiosity to choose a newly-added item and spend more time using the RS.

## 6 Results analysis

The results of testing the old and new evaluation rules with worst-case (random) suggestions directly indicate the effects of unexpected suggestions on the results. The observed variations in the results suggest that these effects are reasonably stable, indicating the importance of controlling the suggestions presented by an RS proportionately to a user's behaviors in regard to diversity or novelty based on the new evaluation rules. For this reason, we propose two approaches for potentially benefiting from the new rules.

**Figure 50:**
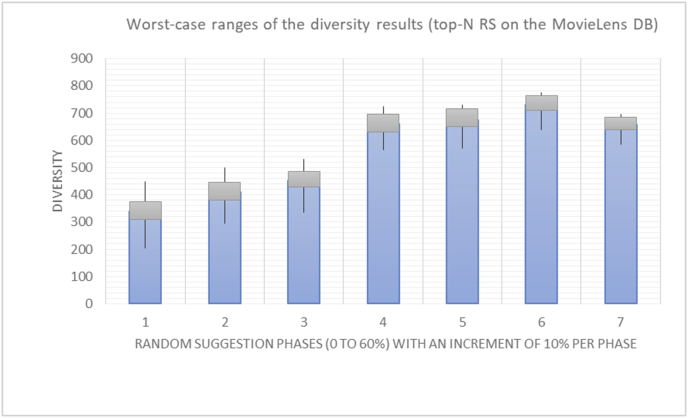
Variation in the results for diversity.

**Figure 51:**
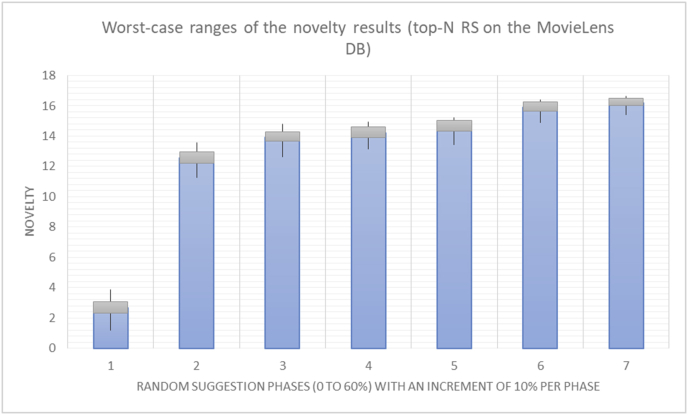
Variation in the results for novelty.

Even with highly unstable results (high variations) when the original RS suggestions are replaced with random (unexpected) suggestions, a pattern of change in the results from one randomness percentage to another can still be identified, especially when the average results are calculated for every level of randomness (Figures 50 and 51).

This sparsity is not a critical concern in practice because there are many choices available in RSs for enriching the diversity and novelty of the results without introducing random selections, such as choosing from among the top 100 choices for diversity enrichment or from a pre-prepared table for novelty enrichment.

**Figure 52:**
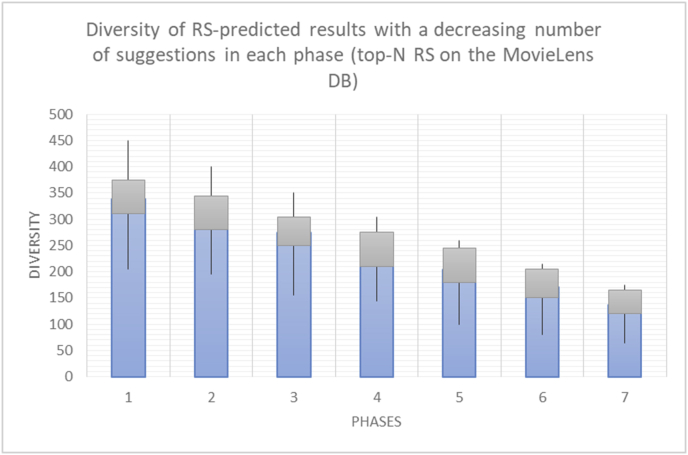
Diversity variations.

**Figure 53:**
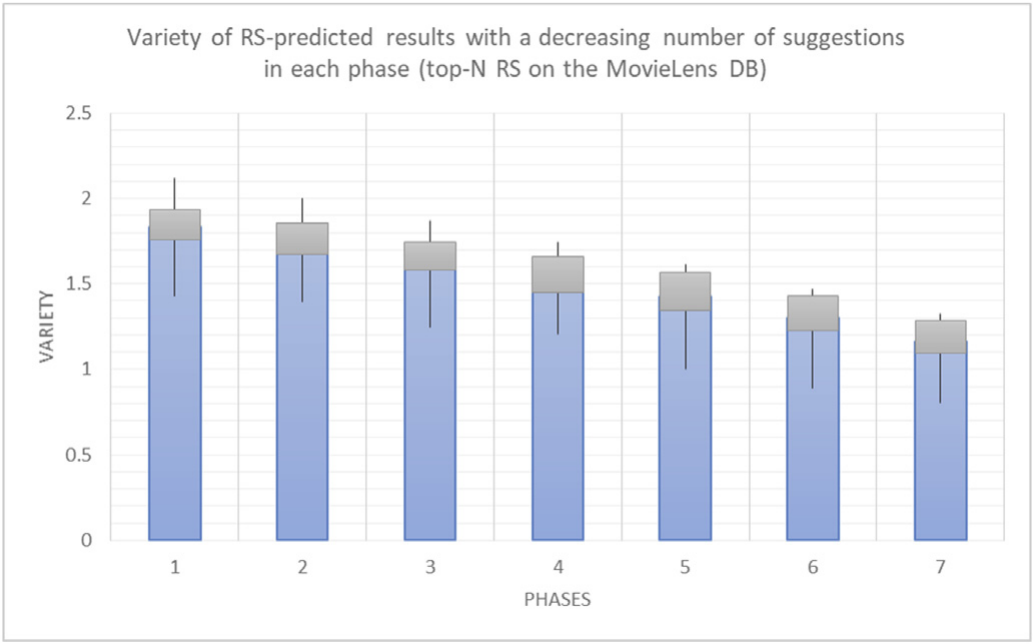
Variety variations.

**Figure 54:**
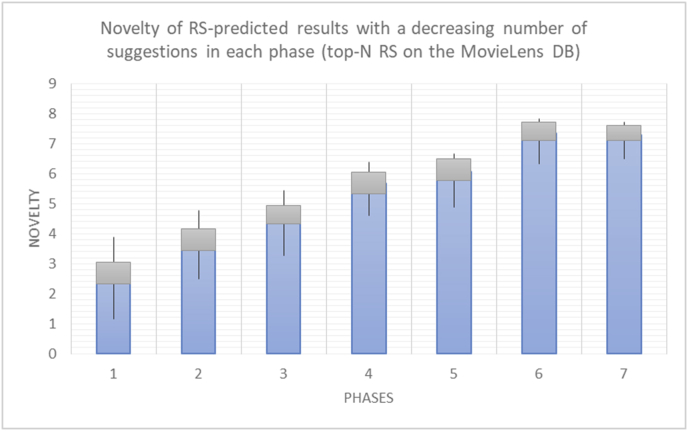
Novelty variations.

**Figure 55:**
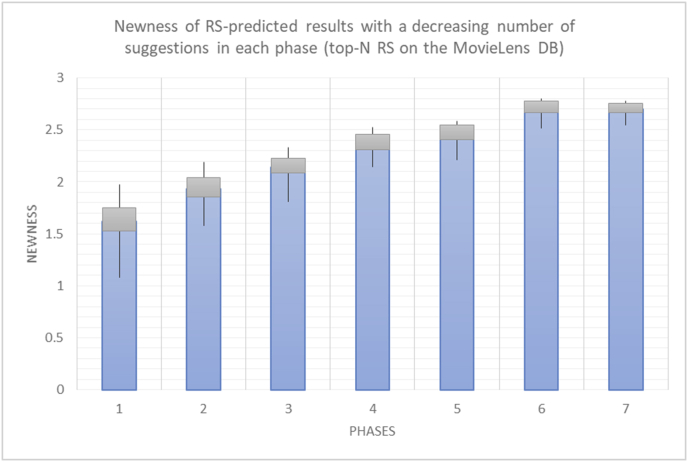
Newness variations.

When considering some of the results from previous experiments (Figures 52, 53, 54, 55) in order to compare the old and new rules (diversity and variety, novelty and newness), one may easily observe that the variety and newness rules are much more accurate and useable than the diversity and novelty rules due to the use of the square root in the new rules, which enables higher weighting and more precise results. Additionally, the new rules are more stable against variations in the inputs. In other words, the new rules yield better confidence intervals because of their centralized and stable results. If the variation in the results or the sparsity of the results is measured in terms of the average frequency of variation between lower and higher values for each rule outcome, the average percentages are 57.29% for diversity and 35.27% for novelty, compared to 34.74% for variety and 20.43% for newness. Thus, if the variety rule is used instead of the diversity rule, an increase of 22.54% in the centralization of the results will be obtained, and 14.84% more centralization will be achieved by using the newness rule instead of the novelty rule (Table 7).

**Table 7:** Diversity, variety, novelty and newness variations in Figures 53, 54, 55, 56.

Phase	Diversity	Variety	Novelty	Newness
	Variation frequency percentage	Variation frequency percentage	Variation frequency percentage	Variation frequency percentage
1	54.44	32.5	70	45.22
2	51.25	30.17	47.67	27.66
3	55.71	33.45	39.79	22.4
4	52.45	31.05	27.82	15.04
5	61.53	37.98	26.66	14.36
6	62.79	39	19.14	10.08
7	62.85	39.05	15.82	8.25
**Average Percentage**:	57.29	34.74	35.27	20.43

The narrower distribution and reduced variation of the results are helpful for achieving greater precision in item evaluations, which can help a RS make better decisions regarding the selection of items when the new rules are used in place of the old rules for the evaluation of the items in the item recommendation list.

### 6.1 Variety approach

Next, a method is presented for ensuring high-quality analysis and applicability of the variety rule.

**Figure 56:**
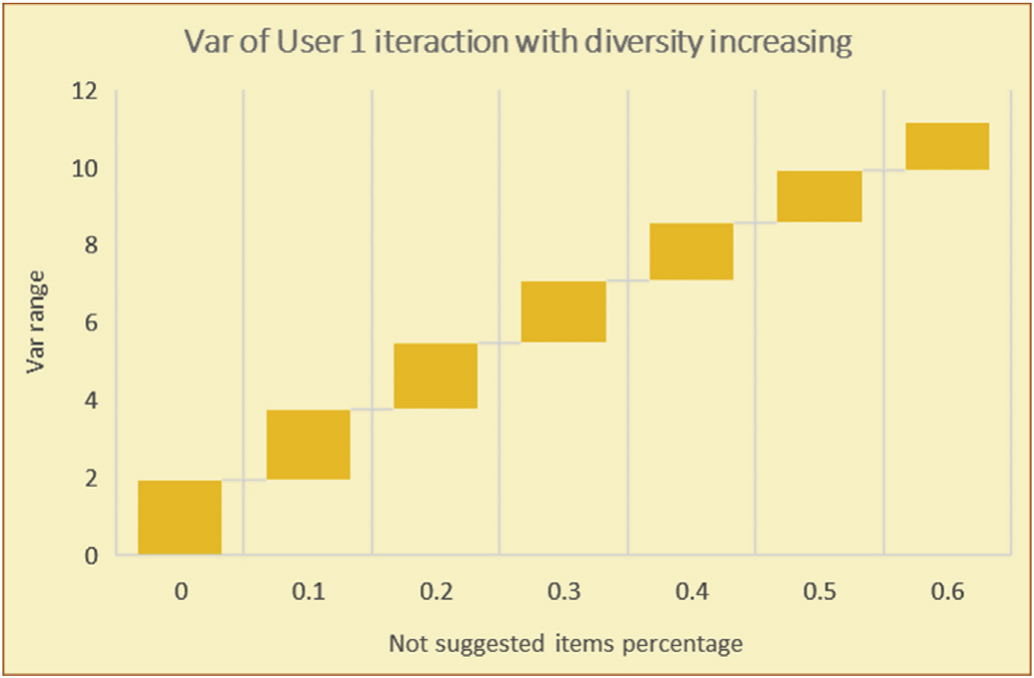
Diagram of variety phases based on the Var value.

In Figure 56, with zero percent unexpected suggestions, testing of the Var value is useless; thus, the discovery of a user's interactions with diversified suggestions must start with the addition of various unpredicted items, at different (cumulative) percentages divided into incremental phases. Then, Var_i_ in the current phase and Var_p_ in the previous phase can be compared.

If Var_i_ < Var_p_, then the user does not like the increased diversity of the suggestions, and it is better to decrease the percentage of diversifications (return to the previous phase).

If Var_i_ > Var_p_, then the user shows good responsiveness to the increased diversity, and it is reasonable to risk a further increase (proceed to the next phase).

### 6.2 Newness approach

Next, a method is presented for ensuring high-quality analysis and applicability of the newness rule.

**Figure 57:**
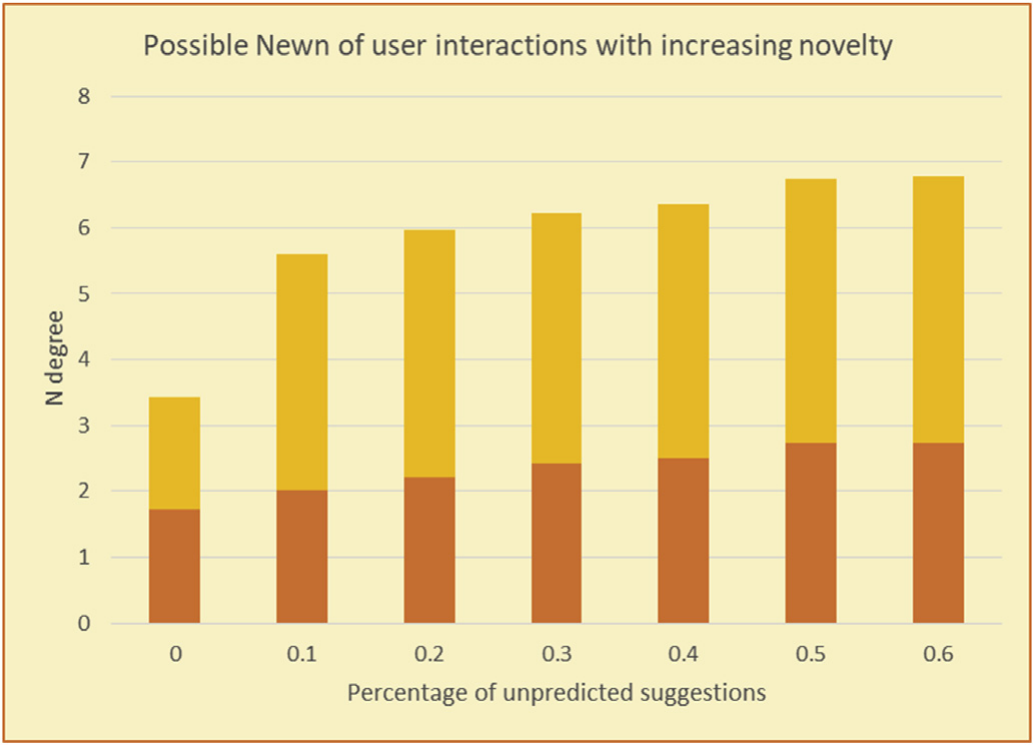
Diagram of newness phases based on the Newn value.

In Figure 57, with zero percent unpredicted new suggestions, testing of the Newn value is useless; thus, discovery of a user's interactions with novel suggestions must start with the addition of new items, different (cumulative) percentages divided into incremental phases. The system compares the user's Newn value with those of the new suggestions and RS suggestions in every phase to determine whether the user responds well to the existence of new items. In simple terms, the user's Newn value is always within the following domain:

RS items' Newn (RSN) < User Newn (UN) < New items’ Newn (NN)

If UN is closer to RSN, then the user has not received any benefit from the new suggestions, and it is better to decrease the percentage of new suggestions (return to the previous phase to decrease the novelty of the suggestions).

If UN is closer to NN, then the user has shown good responsiveness to the new suggestions, and it is reasonable to risk further increasing the percentage of new suggestions (proceed to the next phase to increase the novelty of the suggestions).

### 6.3 Discussion and limitations

According to previous reports, there is an important need to provide new evaluation approaches that are more adaptive to developments in the RS field [8]. One of the most important goals of an RS is to increase user satisfaction and utility rather than to increase accuracy [15, 40]. One way to improve user satisfaction is to better understand user behaviors (perspectives). However, such efforts have not been sufficiently studied [25]; it is only that many research works have begun to show an interest in characterizing user behavior [14] by adding psychological dimensions to RS analysis and filtering methods [26, 39].

The proposed variety and newness rules were developed to solve the problem of controlling suggestions based on user satisfaction measurement; they are limited to the diversity or novelty evaluation of the RS-selected suggestions in the suggestions list, but can also be used in place of previous diversity or novelty rules by evaluating all RS predictions where they provide better results, as explained in the analysis section. New rules succeed to enhance user satisfaction and attract users to spend more time using RS by detecting each user preference. Testing shows that the new rules result in improved RS performance in terms of the high flexibility and response for the user selections behavior. In other words, the research achieved two main goals, first: it introduced two new evaluation rules that can evaluate RS diversity and novelty based on user behavior. Second: The RS will be able to be very adaptive to the user-appropriate novelty or diversity through a real-time response to user-selection evaluations.

The new evaluation rules provide higher efficacy when used within RS algorithms. In addition, they are more practical, logical and effective in phased form (because such phases determine the optimal time slices for calculating the N or Var based on the needs of each RS and user activities). Without the phased approach, such a system is very sensitive to any user interaction. Hence, there is a large risk of losing the desired understanding of user behavior and trust in the system.

In addition, the regulation of the novelty and diversity of the final suggestions will certainly affect their accuracy; that is because introducing more new items (more novelty or unexpectedness) or more diversity will lead to lower accuracy, but it can enable better satisfaction and attractiveness for users based on the new rules evaluations. If an RS deduces from the new evaluations a tendency for novelty or diversity, the adding of the unexpected item (approximately 10 percent lower accuracy in current research) should be done incrementally (parts 6.1 and 6.2) with an evaluation of user reaction with each set of suggestions.

The same applies in the case of user tendency for more accuracy, where the RS should reduce the number of unexpected items in this case (in current research, 10 percent less unexpected items will approximately increase the accuracy by 10 percent). The adding or reducing of unexpected items will test user acceptance of new novelty or diversity, and the RS will understand this acceptance based on the evaluation of a set of consecutive user selections, which provide values (newness and variety) of user tendencies and help the RS to deliver a more accepted suggestions list, leading to more satisfaction and time spent using the RS.

The percentage variation of accuracy (the 10 percent) is changed a little due to each item evaluation in the suggestion list, which requires an evaluating of each selection. Also, accuracy variation between suggestion sets is required to study RS evaluation priorities, even with the better satisfaction achieved by the new rules. So, it is up to the RS to choose whether to modify suggestions based on user behavior or to control this modification by identifying the minimum accepted accuracy that will stop additional replacement of RS suggestions by new ones.

**Figure 58:**
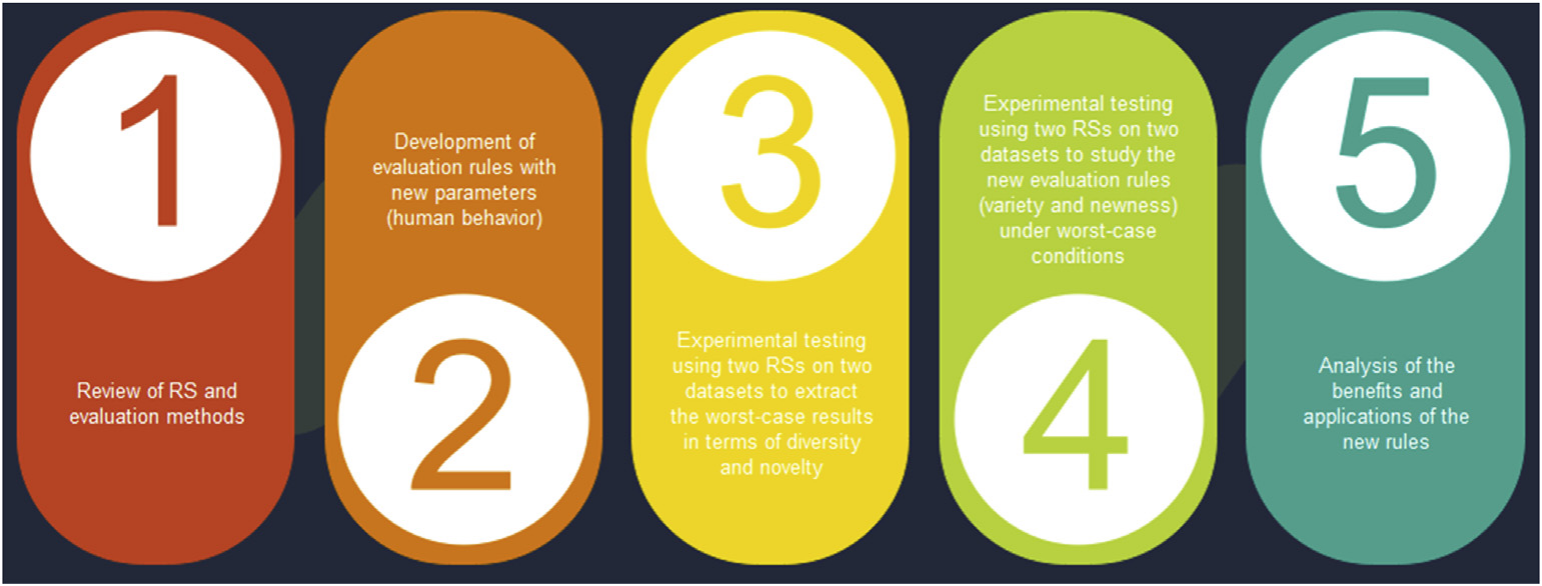
Main research components.

This research presents a new concept of evaluation, which is summarized by adding a new dimension on two evaluation types that can evaluate RS and help pave the way for more efficient RS with more effective results through the real-time evaluation method, and is suggested as a good process for using new rules inside RS (Figure 58: summarizes the entire research workflow).

## 7 Conclusion

The concepts of variety and newness are proposed as the basis for two new evaluation rules considering human behavior to calculate specific quantitative measures that can be used in any RS to adapt the types of suggestions presented. These new rules provide better satisfaction with respect to users’ thought patterns as deduced from their dynamic item selections in separate suggestion phases, where every phase of suggestion generation is independently studied to assess the degrees of variety and newness of the selections.

This research tests the benefits of adding random suggestions to the initial RS-predicted suggestions and then proves the results using the newly proposed evaluation approaches. The new approaches are helpful in testing and monitoring the common predictions of RSs with enhanced variety and may be helpful in the future for incorporating the predicted suggestions of other RSs.

The presented solutions enable higher weighting and centralization for predictions, thus guiding better selection among items with similar evaluation values. This can facilitate the evaluation, analysis and comparison of future similar RS approaches, thus supporting better, faster RS development.

A major innovation for solving the main suggested problem has been presented in a previous survey [6]. Some other surveys have referred to it in an indirect manner. In the future, we plan to extend the present results to fuzzy classes based on fuzzy mathematical methods, which provide easier standard definitions for new RSs. Additionally, we plan to develop solutions in more efficient and practical forms and develop more RS standards. Moreover, we will study the development of new rules for evaluating user behavior with regard to new parameters and the benefits of these rules and will develop new parameters for balancing the relevance of diversity, novelty, variety, newness and accuracy.

## Declarations

### Author contribution statement

M. Kshour: Conceived and designed the experiments; Performed the experiments; Analyzed and interpreted the data; Contributed reagents, materials, analysis tools or data; Wrote the paper.

M. Ebrahimi: Conceived and designed the experiments; Analyzed and interpreted the data; Contributed reagents, materials, analysis tools or data; Wrote the paper.

S. Goliaee and R. Tawil: Analyzed and interpreted the data; Contributed reagents, materials, analysis tools or data; Wrote the paper.

### Funding statement

This research did not receive any specific grant from funding agencies in the public, commercial, or not-for-profit sectors.

### Data availability statement

Data associated with this study has been deposited at https://grouplens.org/datasets/.

### Declaration of interests statement

The authors declare no conflict of interest.

### Additional information

No additional information is available for this paper.
